# PGC1α‐Inducing Senomorphic Nanotherapeutics Functionalized with NKG2D‐Overexpressing Cell Membranes for Intervertebral Disc Degeneration

**DOI:** 10.1002/advs.202400749

**Published:** 2024-03-30

**Authors:** Sheng Liu, Kanglu Li, Yuxin He, Sheng Chen, Wenbo Yang, Xuanzuo Chen, Shiqing Feng, Liming Xiong, Yizhong Peng, Zengwu Shao

**Affiliations:** ^1^ Department of Orthopedics Union Hospital Tongji Medical College Huazhong University of Science and Technology Wuhan 430022 China; ^2^ The Second Hospital of Shandong University Cheeloo College of Medicine Shandong University Jinan 250033 China; ^3^ Department of Orthopedics Qilu Hospital of Shandong University Cheeloo College of Medicine Shandong University Jinan 250012 China; ^4^ Department of Orthopedics Tianjin Medical University General Hospital Tianjin Medical University Tianjin 300052 China

**Keywords:** cell membrane coating, cellular senescence, intervertebral disc degeneration, nanotherapeutic, NKG2D, PGC1α

## Abstract

Cellular senescence is a significant contributor to intervertebral disc aging and degeneration. However, the application of senotherapies, such as senomorphics targeting senescence markers and the senescence‐associated secretory phenotype (SASP), remains limited due to challenges in precise delivery. Given that the natural killer group 2D (NKG2D) ligands are increased on the surface of senescent nucleus pulposus (NP) cells, the NKG2D‐overexpressing NP cell membranes (NNPm) are constructed, which is expected to achieve a dual targeting effect toward senescent NP cells based on homologous membrane fusion and the NKG2D‐mediated immunosurveillance mechanism. Then, mesoporous silica nanoparticles carrying a peroxisome proliferator‐activated receptor‐ɣ coactivator 1α (PGC1α)inducer (SP) are coated with NNPm (SP@NNPm) and it is found that SP@NNPm selectively targets senescent NP cells, and the SP cores exhibit pH‐responsive drug release. Moreover, SP@NNPm effectively induces PGC1α‐mediated mitochondrial biogenesis and mitigates senescence‐associated markers induced by oxidative stress and the SASP, thereby alleviating puncture‐induced senescence and disc degeneration. This dual‐targeting nanotherapeutic system represents a novel approach to delivery senomorphics for disc degeneration treatment.

## Graphical abstract

This work illustrates a biomimetic dual‐targeting strategy to deliver senomorphics to senescent cells. Exhibiting cell membrane homology and the immunosurveillance receptor‒ligand interactions, the NKG2D‐overexpressing nucleus pulposus cell membranes enhance the targeting ability of the mesoporous silica nanoparticles loaded with PGC1α inducers. The nanotherapeutic approach that enhances PGC1α‐mediated mitochondrial biogenesis can alleviate mitochondrial dysfunction, cellular senescence, and intervertebral disc degeneration.

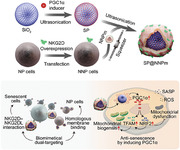

## 1 Introduction

Intervertebral disc degeneration (IVDD) is a major cause of low back pain that decreases quality of life and causes a substantial socioeconomic burden.^[^ ^1^ ^]^ The current treatment options for disc degeneration include conservative approaches involving pain medications and surgical discectomy procedures; however, these treatments remain ineffective.^[^ ^2^ ^]^ Therefore, there is an urgent need to develop therapies with potent activity and desirable properties for IVDD treatment.

Cellular senescence is a major driving force of disc aging and degeneration.^[^ ^3^ ^]^ Senescent cells exhibit irreversible cell cycle arrest, cell cycle checkpoint regulator dysregulation (e.g., p21 and p16), cellular metabolism abnormalities, and the senescence‐associated secretory phenotype (SASP). The SASP results in the production of high levels of proinflammatory molecules and extracellular matrix‐degrading enzymes, which are critical factors underlying IVDD development.^[^ ^4^, ^5^ ^]^ There are two main strategies for targeting senescence: using senolytics to kill senescent cells by targeting anti‐apoptosis pathways and using senomorphics to suppress senescence markers, particularly the SASP.^[^ ^6^ ^]^ Recently, long‐term administration of the senolytic drugs Dasatinib and Quercetin has been shown to ameliorate IVDD.^[^ ^7^ ^]^ However, the off‐target toxicity of senolytics may limit their clinical application.^[^ ^8^, ^9^ ^]^ Several studies have suggested that bioactive materials could attenuate IVDD by inhibiting cellular senescence without causing cell death.^[^ ^10^, ^11^ ^]^ Mitochondrial dysfunction and consequent redox imbalance are shared hallmarks of cellular senescence and IVDD, yet translational solutions to address these issues are limited.^[^ ^12^, ^13^, ^14^ ^]^ Thus, developing more bioavailable senomorphics with mitochondrion‐targeted or redox‐modulating properties for IVDD treatment would be prudent.

Emerging evidence suggests that mechanisms related to mitochondrial quality control, such as mitochondrial biogenesis, are involved in cellular senescence and aging.^[^ ^15^ ^]^ Peroxisome proliferator‐activated receptor‐ɣ coactivator 1α (PGC1α), which acts as a master regulator of mitochondrial biogenesis and redox homeostasis, activates the transcription factor nuclear respiratory factor‐1/2 (NRF1/2) and thereby controls the expression of mitochondrial transcription factor A (TFAM).^[^ ^16^, ^17^ ^]^ Several studies have shown that PGC1α is downregulated in degenerative discs and participates in the regulation of cellular senescence during IVDD.^[^ ^18^, ^19^, ^20^ ^]^ Fortunately, a novel benzimidazole compound, ZLN005, has been identified as a transcriptional inducer of PGC1α and has therapeutic effects in diseases involving PGC1α downregulation.^[^ ^21^ ^]^ Given the protective role of PGC1α upregulation in aging,^[^ ^22^ ^]^ whether this PGC1α inducer (Pi, ZLN005) can effectively protect against IVDD as a senomorphic drug deserves further study.

Biomimetic strategies can enhance the advantages of synthesized nanoparticles, including high specificity and biocompatibility, through existing biological pathways or structures.^[^ ^23^ ^]^ Previous research aiming to enhance the senescence‐targeting ability of nanoparticles utilized only senescence markers, including the substrate of senescence‐associated β‐galactosidase (SA‐β‐Gal), lactose‐linked CD9 monoclonal antibodies, or molecularly imprinted nanoparticles targeting B2M extracellular epitopes.^[^ ^24^, ^25^, ^26^ ^]^ A recent study illustrated that rapid penetration and organelle‐level delivery into chondrocytes could be achieved through camouflage encapsulation and membrane fusion mediated by cellular membranes.^[^ ^27^ ^]^ Notably, nanoparticle delivery efficacy could be enhanced by coating them with homologous cell membranes and incorporating cell‐specific receptors or elements.^[^ ^28^, ^29^ ^]^ The receptor expressed on the surface of cytotoxic immune cells, natural killer group 2D (NKG2D), binds various cell surface ligands, mediating immune surveillance and the clearance of abnormal cells, including senescent cells.^[^ ^30^, ^31^ ^]^ These ligands, such as MHC class I polypeptide–related sequence A/B (MICA/MICB) and retinoic acid early transcript 1E (RAET1E), are collectively referred to as NKG2D ligands (NKG2DLs) and can be upregulated by various senescence‐associated stresses.^[^ ^31^, ^32^ ^]^ Recently, NKG2D in the immune cells has been shown able to recognize the upregulated NKG2DLs on senescent cells and serve as a selective senolytic agent for aging and age‐related diseases.^[^ ^33^ ^]^ However, whether the efficacy of nanoparticles targeting senescence can be enhanced by a dual‐targeting strategy, combining a homologous membrane coating and the NKG2D–NKG2DL interactions, needs further investigation.

**Figure 1:**
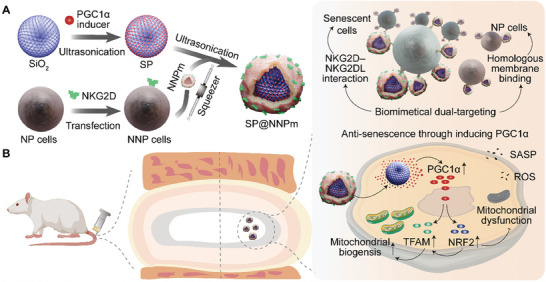
Schematic illustration of the preparation of dual‐targeting nanoplatform and its principle of protecting efficacy by delivering a PGC1α inducer (Pi) for IVDD. A) The preparation procedures of nanoparticles. The mesoporous SiO_2_ nanoparticles were ultrasonicated with the Pi to generate Pi‐loaded SiO_2_ nanoparticles (SP). After harvesting cell membranes from NKG2D‐overexpressing NP (NNP) cells, membrane coating was performed to generate SP coated with NNP cell membranes (SP@NNPm) by ultrasonication and physical co‐extrusion using a nano‐squeezer. B) Nanoparticles functionalized with cell membrane coating have the dual‐targeting ability to senescent NP cells in the intervertebral disc. NP cell membrane selectively fuses with homologous cells. NKG2D receptor recognizes NKG2D ligands (NKG2DLs) expressed on the surface of cells undergoing senescence. The Pi increases the expression of PGC1α and its target genes NRF2 and TFAM, thereby promoting mitochondrial biogenesis and alleviating redox imbalance in ROS or SASP‐induced senescence. PGC1α, peroxisome proliferator‐activated receptor‐ɣ coactivator 1α; NKG2D, the natural killergroup 2D; NRF2, nuclear respiratory factors‐2; TFAM, mitochondrial transcription factor A; ROS, reactive oxygen species; SASP, senescence‐associated secretory phenotype. The rat icon was created with BioRender.com.

Here, we developed a dual‐targeting nanoplatform referred to as SP@NNPm for targeting cellular senescence in IVDD. In brief, NKG2D‐overexpressing nucleus pulposus (NP) cell membranes (NNPm) were coated with the stellate monodisperse mesoporous silica nanoparticles (SiO_2_) to deliver the Pi into NP cells (**Figure** **1**). The design offers the following advantages: (I) the pH‐responsive release of a PGC1α inducer from silica nanospheres enhances the selective promotion of mitochondrial biogenesis and antioxidant genes, effectively countering the effects of cellular senescence and the SASP; (II) the homotypic fusing ability of the NP cell membrane maintains excellent extracellular stability and facilitates uptake by NP cells, making it more suitable for intervertebral disc applications; and (III) the recognition of the upregulated NKG2DLs in senescent cells enhances the targeting efficacy toward senescent NP cells. Overall, this study presents a biomimetic targeting strategy that holds promise for delivering senomorphics to address mitochondrial dysfunction and redox imbalance in IVDD.

## 2 Results

### 2.1 Design, Preparation, and Characterization of Dual‐Targeting Nanoparticles

**Figure 2:**
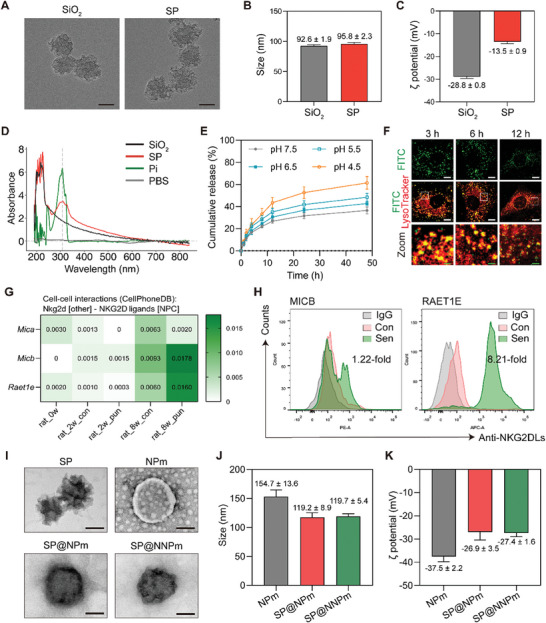
Preparation and characterization of the dual‐targeting nanoplatform. A) Scanning electron microscopy images of the prepared nanoparticles SiO_2_ and SP. Bar = 50 nm. B,C) Barplots of particle sizes (B) and zeta potential (C) of the nanoparticles SiO_2_ and SP (*n* = 3). Data are presented as the mean ± standard deviation (SD). D) The UV spectrum for the prepared Pi, SiO_2_, and SP. PBS was used as the control. The Pi peaks were indicated with dashed lines at ≈310 nm. E) Dissolution curve of the Pi in SP nanoparticles in PBS with a pH range of 4.5–7.5 (*n* = 3). Data are presented as the mean ± SD. F) Representative confocal images of NP cells exposed to LysoTracker and FITC‐doped SP for 3, 6, or 12 h. White bar = 5 µm; Green bar = 0.8 µm. G) The heatmap plot for the predicted interaction strength between NKG2D of other cells and NKG2D ligands (MICA/*Mica*, MICB/*Micb*, RAET1E/*Raet1e*) of NPC based on the CellPhoneDB database. The cell–cell interaction analysis was based on the merged datasets GSE154884 (generated from disc cells of 8‐week‐old rats, defined as rat_0w) and GSE211407 (generated from disc cells of rats undergoing puncture‐induced IVDD or in a sham group for 2 weeks or 8 weeks, grouped as rat_2w_con, rat_2w_pun, rat_8w_con, and rat_8w_pun). The color indicated the mean of interaction strength in samples of the five groups. H) Representative flow cytometry plot for MICB and RAET1E expressions on the cellular surface of senescent cells (Sen) and control cells (Con). IgG represents isotype control antibody staining. Fold change was defined as the mean fluorescence intensity ratio (Sen/Con). I) Transmission electron microscopy images of the prepared nanoparticles SP, NPm, SP@NPm, and SP@NNPm. Bar = 50 nm. J,K) Barplots of particle sizes (J) and zeta potential (K) of NPm, SP@NPm, and SP@NNPm (*n* = 3). Data are presented as the mean ± SD. Pi, PGC1α inducer; SP, the PGC1α inducer‐loaded SiO_2_; PBS, phosphate buffered saline; NPC, NP cells; other, other cell; FITC, fluorescein isothiocyanate; NPm, NP cell membranes; NKG2DLs, NKG2D ligands; NNP, NKG2D‐overexpressing cells; SP@NPm, SP coated with NPm; SP@NNPm, SP coated with NKG2D‐overexpressing NPm.

Mesoporous SiO_2_ nanoparticles were synthesized and loaded with the Pi using ultrasonication for controlled release. Scanning electron microscopy suggested that the prepared SiO_2_ nanoparticles exhibited a similar spherical morphology with mesoporous structures (**Figure** **2**A). Dynamic light scattering revealed similar average diameters of the nanoparticles, while zeta potential analysis revealed an increase in the zeta potential of the SP compared to that of SiO_2_ (Figure 2B,C). The presence of a Pi peak at ≈310 nm in the UV–vis spectrum of SP not present in SiO_2_ further confirmed the successful synthesis of the drug‐loaded nanoparticles (Figure 2D). Drug‐loading assays of the SiO_2_ nanoparticles revealed that the loading content was 2.12% ± 0.12%, and the loading efficiency was 63.4% ± 3.13%. Next, we evaluated the cumulative release profile of the Pi from SP at several pH levels (pH 4.5–7.5), given the significance of lower intracellular pH and increased lysosomal mass in the senescence process.^[^ ^34^ ^]^ The results indicated that ≈50% of the drug was released within 48 h (Figure 2E). Specifically, SP exhibited a higher release rate in an acidic environment resembling intracellular conditions (pH 4.5) than at a neutral pH (pH 7.5). Moreover, confocal images of NP cells treated with fluorescein isothiocyanate (FITC)‐doped SP and LysoTracker probes revealed that the green (FITC fluorescence) and the red (LysoTracker fluorescence) signals in lysosomes merged well into yellow at 3 h, and the fluorescence gradually decreased at 6–12 h, indicating the accumulation and decomposition of SP in lysosomes (Figure 2F). These findings suggest the successful synthesis of SP cores with high loading efficiency and pH‐responsive release behavior.

Cell membrane coating is recognized as an attractive nanotechnology in the rational design of biomimetic nanoparticles.^[^ ^35^ ^]^ To develop a biomimetic strategy, we analyzed the profiles of membrane proteins and intercellular communication during puncture‐induced IVDD using merged single‐cell ribonucleic acid (RNA) sequencing datasets (Figure S1A, Supporting Information). Cell identify analysis revealed that NP cells expressed aggrecan (*Acan*) and the collagen type II alpha 1 chain (*Col2a1*) (*Acan*/*Col2a1* > 2), and membrane proteins such as CD24, CD155 (*poliovirus receptor* [*Pvr*]), CD221 (*insulin‐like growth factor 1 receptor* [*Igf1r*]), and interleukin 6 receptor (*Il6r*) (Figure S1B, Supporting Information). CellChat analysis revealed that puncture treatment decreased the number and strength of interactions among disc cells, especially between NP cells (Figure S1C,D, Supporting Information). In addition, the receptor–ligand analysis using the CellPhoneDB database illustrated that the strength of the interaction between NKG2DLs, including MICB (*Micb*) and RAET1E (*Raet1e*), of NP cells and the NKG2D of the identified other cells, was increased at 8 w post‐puncture (Figure 2G). To investigate the change of membrane NKG2DL expression in senescent NP cells, we treated NP cells with tert‐butyl hydroperoxide (TBHP), a typical inducer of cellular senescence.^[^ ^36^ ^]^ Sublethal exposure to TBHP (70 µmol^ ^L^−1^ for 3 h) resulted in the senescence of most NP cells, as evidenced by the growth ability loss and positive staining for SA‐β‐Gal (Figure S2A–C, Supporting Information). Thus, senescent NP cells were harvested for further experiments. Flow cytometry assays revealed that the expression of MICB and RAET1E was higher on the cell surface of senescent NP cells than on the surface of control cells (Figure 2H). These results indicate that combining the NKG2D–NKG2DL interaction with the NP cell membrane may be a suitable candidate for membrane coating to promote the recognition of senescent NP cells.

We next transfected plasmids into NP cells (Figure S3A–D, Supporting Information) and generated cell membranes from empty plasmid‐transfected NP cells (NPm) or NNPm to coat the SP cores (SP@NPm and SP@NNPm, respectively). Transmission electron microscopy revealed that the SP cores were successfully encapsulated by NPm or NNPm after physical coextrusion (Figure 2I). Drug‐loading assays indicated that the encapsulation efficiency was 82.7% ± 1.99% for SP@NPm and 83.4% ± 1.32% for SP@NNPm. SP@NPm and SP@NNPm displayed similar average sizes and zeta potentials (Figure 2J,K). These results demonstrate the successful establishment of an SP@NNPm nanoplatform with dual‐targeting potential.

### 2.2 Targeting Effects of SP@NNPm on Senescent NP Cells

**Figure 3:**
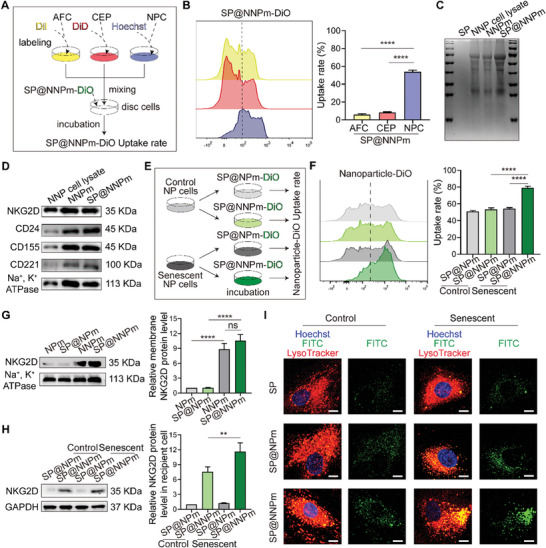
Enhancement of nanoparticle targeting to senescent cells by NKG2D‐overexpressing NP cell membranes. A) Schematic for assays evaluating the targeting ability of SP@NNPm. SP@NNPm labeled with DiO was administrated after labeling and mixing disc cells. B) Representative plots (left) and statistical analysis (right, *n* = 3) of flow cytometry assays indicating the specific uptake of SP@NNPm‐DiO by disc cells. Data are presented as the mean ± SD, ^****^ *p* < 0.0001 between groups. C) Representative Coomassie blue staining plot indicating the membrane protein spectrum for the SiO_2_, NNP cell lysate, NNPm, and SP@NNPm. D) Representative Western blotting plots indicating the NP cell membrane proteins CD24, CD155, CD221, and NKG2D in the NNP cell lysate, NNPm, or SP@NNPm. E) Schematic for assays evaluating the targeting ability of nanoparticles labeled with DiO on control and senescent NP cells. F) Representative flow cytometry plots (left) and statistical analysis (right, *n* = 3) of the uptake of SP@NPm‐DiO or SP@NNPm‐DiO by senescent or control NP cells. Data are presented as the mean ± SD, ^****^ *p* < 0.0001 between groups. (G) Representative Western blotting plots (left) and statistical analysis (right, *n* = 3) of membrane proteins in NPm, SP@NPm, NNPm, and SP@NNPm. Na^+^, K^+^ ATPase was defined as membrane control. Data are presented as the mean ± SD, ns, no significance, ^****^ *p* < 0.0001 between groups. H) Representative Western blotting plots (left) and statistical analysis (right, *n* = 3) of NKG2D levels in senescent or control NP cells treated with SP@NPm or SP@NNPm. Data are presented as the mean ± SD, ^**^ *p* < 0.01 between groups. I) Representative confocal images showing the uptake and distribution of FITC‐doped nanoparticles by senescent or control NP cells stained with Hoechst and LysoTracker. Bar = 5 µm. AFC, annulus fibrosus cells; CEP, cartilaginous endplate cells; NPC, NP cells; NNP cell, NKG2D‐overexpressing NP cell; NNPm, NKG2D‐overexpressing NP cell membranes; SP@NPm, SP coated with NP cell membranes; SP@NNPm, SP coated with NKG2D‐overexpressing NP cell membranes.

Disc cells prestained with different dyes were coincubated with DiO‐labeled SP@NNPm (SP@NNPm‐DiO) to evaluate the cell type‐targeting specificity of SP@NNPm (**Figure** **3**A). Flow cytometry assays revealed that SP@NNPm‐DiO mostly targeted NP cells (Figure 3B). Coomassie blue staining of SP@NNPm confirmed the retention of cell membrane characteristics, as evidenced by a membrane protein pattern like that of NNPm (Figure 3C). Western blotting of SP@NNPm revealed positivity for several NP cell membrane markers including CD24, CD155, and CD221 (Figure 3D), in which cellular homology may explain the homotypic targeting ability of SP@NNPm.^[^ ^37^, ^38^ ^]^ Next, control and senescent NP cells were treated with SP@NNPm‐DiO or SP@NPm‐DiO to test the affinity of the nanoparticles for senescent cells (Figure 3E). Flow cytometry assays revealed that senescent NP cells exhibited a greater uptake rate of SP@NNPm than control NP cells, and SP@NNPm exhibited a greater affinity for senescent NP cells than SP@NPm (Figure 3F). Western blotting revealed that SP@NNPm maintained the NKG2D positivity in NNPm (Figure 3G). Furthermore, SP@NNPm treatment resulted in increased NKG2D levels in senescent cells compared with those in control cells (Figure 3H), confirming the increased uptake of SP@NNPm by senescent cells. Confocal fluorescence microscopy revealed that SP@NNPm could be selectively internalized by senescent NP cells and located in lysosomes (Figure 3I). Taken together, these results suggest the selective dual‐targeting ability of SP@NNPm on senescent NP cells due to cellular homology and NKG2D overexpression on NNPm.

### 2.3 Biocompatibility of SP@NNPm

**Figure 4:**
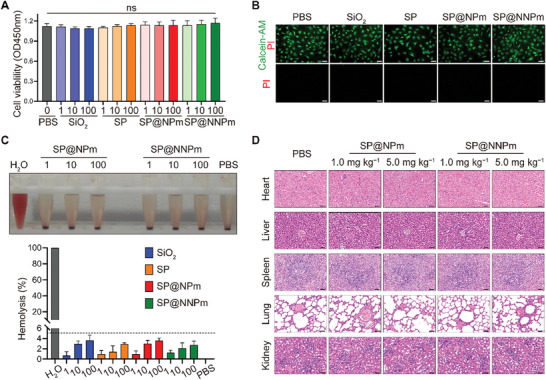
Biocompatibility of nanoparticles. A) Cell viability assays of NP cells exposed to different concentrations (1–100 µg^ ^mL^−1^) of nanoparticles (SiO_2_, SP, SP@NPm, and SP@NNPm) for 24 h (*n* = 3). Data are presented as the mean ± SD, ns, no significance. B) Representative calcein‐AM/propidium iodide staining plot of NP cells treated with 100 µg^ ^mL^−1^ nanoparticles for 24 h. Bar = 50 µm. C) Hemolysis assay on rat red blood cells treated with different concentrations of nanoparticles. The upper image shows representative images for SP@NPm and SP@NNPm. The lower image shows the absorbance value (*n* = 3) at 541 nm of the supernatant after a 12‐hour incubation. Data are presented as the mean ± SD. D) Representative HE staining plot of organs (heart, liver, spleen, lung, kidney) after intravenous injection with different doses of SP@NPm and SP@NNPm for 14 days. Bar = 100 µm. PBS, phosphate buffered saline; PI, propidium iodide; SP, the PGC1α inducer‐loaded SiO_2_; SP@NPm, SP coated with NP cell membranes; SP@NNPm, SP coated with NKG2D‐overexpressing NP cell membranes.

The biocompatibility of the nanoparticles with NP cells was evaluated using the CCK‐8 assays. SiO_2_, SP, SP@NPm, and SP@NNPm at different concentrations (1, 10, and 100 µg^ ^mL^−1^) did not cause a significant decrease in cell viability (**Figure** **4**A). Moreover, calcein‐AM/propidium iodide staining confirmed the nanoparticles' biocompatibility with NP cells (Figure 4B). We next assessed the toxicity of SP@NNPm to other tissues, given the risk of leakage after intradiscal injection.^[^ ^39^ ^]^ Hemolysis assays revealed that nanoparticles at different concentrations were biocompatible with red blood cells (hemolysis rate < 5%) (Figure 4C). Moreover, hematoxylin and eosin (HE) staining suggested that major organs, such as the heart, liver, spleen, lung, and kidney, were not markedly damaged after intravenous administration injection of SP@NPm or SP@NNPm (Figure 4D). Thus, SP@NNPm exhibits in vivo and in vitro biocompatibility.

### 2.4 Efficacy of SP@NNPm on Senescent Cells

**Figure 5:**
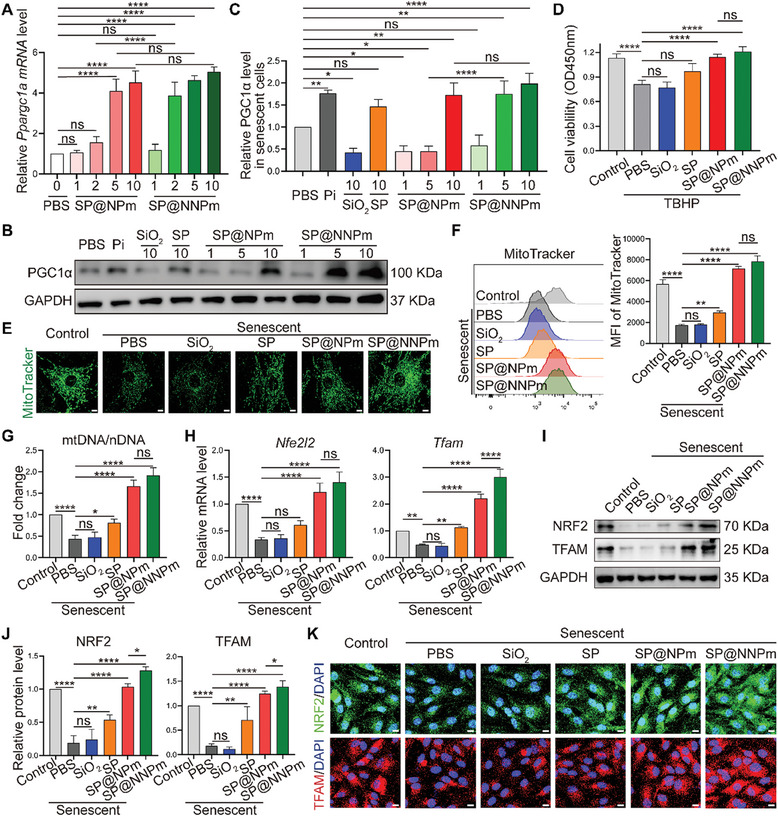
PGC1α‐inducing effect of SP@NNPm on senescent NP cells. A) Statistical analysis of relative expression levels of *Ppargc1a* mRNA in senescent NP cells exposed to SP@NPm or SP@NNPm at different concentrations (1–10 µg^ ^mL^−1^) for 24 h (*n* = 3). Data are presented as the mean ± SD, ns, no significance, ^****^ *p* < 0.0001 between groups. B,C) Representative Western blotting plots (B) and statistical analysis (C, *n* = 3) of PGC1α levels in senescent NP cells exposed to nanoparticles SiO_2_, SP, SP@NPm, or SP@NNPm (1–10 µg^ ^mL^−1^). Pi (5 µg mL^−1^) served as the positive control. Data are presented as the mean ± SD, ns, no significance, ^*^ *p* < 0.05, ^**^ *p* < 0.01, ^****^ *p* < 0.0001 between groups. D) Cell viability assays of normal NP cells treated with 10 µg mL^−1^ nanoparticles and 70 µmol^ ^L^−1^ TBHP for 24 h (*n* = 3). Data are presented as the mean ± SD, ns, no significance, ^****^ *p* < 0.0001 between groups. E,F) Representative confocal images (E) and flow cytometry assays (F, *n* = 3) for MitoTracker staining of NP cells treated with 10 µg^ ^mL^−1^ nanoparticles. Bar = 5 µm. Data are presented as the mean ± SD, ns, no significance, ^**^ *p* < 0.01, ^****^ *p* < 0.0001 between groups. G) Statistical analysis (*n* = 3) of mtDNA/nDNA ratio in senescent NP cells exposed to 10 µg^ ^mL^−1^ SiO_2_, SP, SP@NPm, or SP@NNPm. Data are presented as the mean ± SD, ns, no significance, ^*^ *p* < 0.05, ^**^ *p* < 0.01, ^****^ *p* < 0.0001 between groups. H) Statistical analysis (*n* = 3) of relative expression levels of *Nfe2l2* and *Tfam* mRNA in senescent NP cells exposed to 10 µg^ ^mL^−1^ SiO_2_, SP, SP@NPm, or SP@NNPm. Data are presented as the mean ± SD, ns, no significance, ^**^ *p* < 0.01, ^****^ *p* < 0.0001 between groups. I,J) Representative Western blotting plots (I) and statistical analysis (J, *n* = 3) for NRF2 and TFAM levels in NP cells exposed to 10 µg^ ^mL^−1^ SiO_2_, SP, SP@NPm, or SP@NNPm (*n* = 3). Data are presented as the mean ± SD, ns, no significance, ^*^ *p* < 0.05, ^**^ *p* < 0.01, and ^****^ *p* < 0.0001 between groups. K) Representative images of immunofluorescence staining for NRF2 and TFAM in NP cells exposed to 10 µg^ ^mL^−1^ SiO_2_, SP, SP@NPm, or SP@NNPm. Bar = 25 µm. MFI, mean fluorescence intensity; mtDNA, mitochondrial DNA; nDNA, nuclear DNA; SP, the PGC1α inducer‐loaded SiO_2_; SP@NPm, SP coated with NP cell membranes; SP@NNPm, SP coated with NKG2D‐overexpressing NP cell membranes.

Senescent NP cells were treated with SP@NPm or SP@NNPm at different concentrations and the expression of PGC1α messenger RNA (mRNA) *Ppargc1a* was examined to determine the optimal concentration of nanoparticles for use as senomorphics. Real‐time quantitative polymerase chain reaction (RT‒qPCR) results showed that compared with the phosphate‐buffered saline (PBS) group, the SP@NPm (5–10 µg^ ^mL^−1^) and SP@NNPm (2–10 µg^ ^mL^−1^) treatment groups exhibited significantly increased *Ppargc1a* mRNA levels in senescent NP cells (**Figure** **5**A). Western blotting revealed that treatment of 10 µg^ ^mL^−1^ SP@NPm or SP@NNPm effectively increased PGC1α expression in senescent NP cells (Figure 5B,C). Furthermore, the cell viability of NP cells improved after treatment with 10 µg^ ^mL^−1^ SP, SP@NPm, or SP@NNPm in the presence of TBHP for 24 h, indicating the ability of 10 µg^ ^mL^−1^ nanoparticles to protect against oxidative stress (Figure 5D). Thus, we choose 10 µg^ ^mL^−1^ as the optimal concentration of nanoparticles. MitoTracker staining and mitochondrial DNA/nuclear DNA (mtDNA/nDNA) ratio assays were performed to investigate the effect of 10 µg^ ^mL^−1^ nanoparticles on mitochondrial biogenesis in senescent NP cells. Confocal imaging and flow cytometry assays both demonstrated that compared to other nanoparticles, SP@NPm and SP@NNPm significantly increased the MitoTracker fluorescence intensity in senescent cells (Figure 5E,F), indicating an increase in mitochondrial mass by inducing PGC1α. Furthermore, mtDNA/nDNA ratio assays confirmed the increase in the mtDNA level induced by SP@NPm and SP@NNPm in senescent NP cells (Figure 5G). NRF2 (*Nfe2l2*) and TFAM (*Tfam*), transcription factors regulated by PGC1α, regulate genes associated with mitochondrial biogenesis.^[^ ^40^ ^]^ The RT‒qPCR results showed that the mRNA expression levels of *Nfe2l2* and *Tfam* mRNA in senescent NP cells were significantly increased by SP@NPm and SP@NNPm (Figure 5H). Western blotting and immunofluorescence staining for TFAM and NRF2 confirmed that SP@NPm and SP@NNPm both significantly increased the protein levels of TFAM and NRF2 in senescent NP cells (Figure 5I–K). Thus, SP@NNPm could induce the expression of PGC1α and its target genes NRF2 and TFAM, thereby promoting mitochondrial biogenesis in senescent NP cells.

### 2.5 SP@NNPm Alleviates Cellular Senescence and Dysfunction of NP Cells

**Figure 6:**
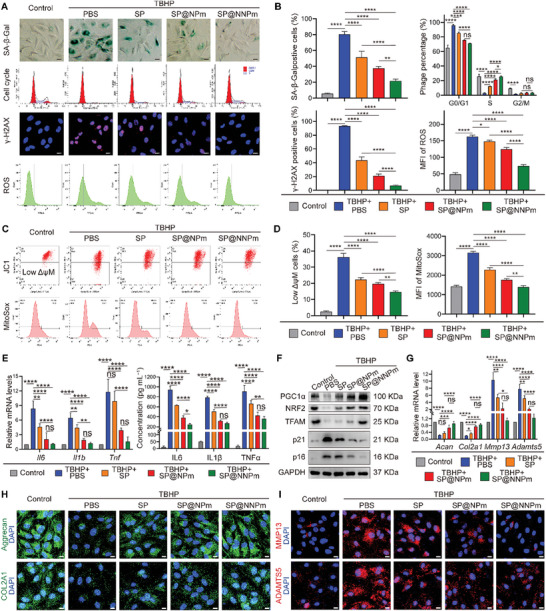
SP@NNPm alleviates cellular senescence and dysfunction of NP cells in vitro. A,B) Representative images (A) and statistical analysis (B) for SA‐β‐Gal staining, cell cycle assays, γ‐H2AX immunofluorescence staining, and ROS staining of NP cells treated with or without 70 µmol^ ^L^−1^ TBHP or 10 µg^ ^mL^−1^ nanoparticles (*n* = 3). Black bar = 50 µm; White bar = 25 µm. Data are presented as the mean ± SD, ns, no significance, ^*^ *p* < 0.05, ^**^ *p* < 0.01, and ^****^ *p* < 0.0001 between groups. C,D) Representative flow cytometry plots (C) and statistical analysis (D, *n* = 3) for JC1 staining and MitoSox staining of NP cells treated with or without 70 µmol^ ^L^−1^ TBHP or 10 µg^ ^mL^−1^ nanoparticles. JC1 aggregates with red fluorescence indicate normal membrane potential, while JC1 probes become monomers with green fluorescence in cells of decreased membrane potential (low ΔψM). Data are presented as the mean ± SD, ^**^ *p* < 0.01, ^****^ *p* < 0.0001 between groups. E) (left) Statistical analysis (*n* = 3) for *Il6*, *Il1b*, and *Tnf* mRNA levels in NP cells treated with or without 70 µmol^ ^L^−1^ TBHP or 10 µg^ ^mL^−1^ nanoparticles. (right) Statistical analysis (*n* = 3) for IL6, IL1β, and TNFα in a conditioned medium of NP cells treated with or without 70 µmol^ ^L^−1^ TBHP or the nanoparticles. Data are presented as the mean ± SD, ns, no significance, ^*^ *p* < 0.05, ^**^ *p* < 0.01, and ^****^ *p* < 0.0001 between groups. F) Representative Western blotting plots showing the levels of PGC1α, p21, p16, NRF2, and TFAM in NP cells treated with or without 70 µmol^ ^L^−1^ TBHP or 10 µg^ ^mL^−1^ nanoparticles. G) Statistical analysis for *Acan*, *Col2a1*, *Mmp13*, and *Adamts5* mRNA levels in NP cells treated with or without 70 µmol^ ^L^−1^ TBHP or 10 µg^ ^mL^−1^ nanoparticles (*n* = 3). Data are presented as the mean ± SD, ns, no significance, ^*^ *p* < 0.05, ^**^ *p* < 0.01, ^***^ *p* < 0.001, and ^****^ *p* < 0.0001 between groups. H,I) Representative immunofluorescence staining plot for Aggrecan, COL2A1, MMP13, and ADAMTS5 in NP cells treated with or without 70 µmol^ ^L^−1^ TBHP or 10 µg^ ^mL^−1^ nanoparticles. Bar = 25 µm. MFI, mean fluorescence intensity. SP, the PGC1α inducer‐loaded SiO_2_; SP@NPm, SP coated with NP cell membranes; SP@NNPm, SP coated with NKG2D‐overexpressing NP cell membranes.

NP cells were treated with 70 µmol^ ^L^−1^ TBHP and 10 µg^ ^mL^−1^ nanoparticles and subjected to senescence‐associated analysis to investigate the senomorphic impact of the nanoparticles. These analyses included SA‐β‐Gal staining, cell cycle assays, immunofluorescence staining for the DNA damage marker gamma‐histone H2A family member X (γ‐H2AX), and staining with the reactive oxygen species (ROS) probe dichlorofluorescein diacetate. Compared with the other nanoparticles, SP@NNPm significantly reduced the TBHP‐increased percentage of SA‐β‐Gal‐positive, G1/S‐arrested, and γ‐H2AX‐positive cells, and decreased the intracellular ROS levels increased by TBHP (**Figure** **6**A,B). Considering the critical role of mitochondrial dysfunction in senescence, we investigated the mitochondrial membrane potential (ΔψM) and mitochondrial ROS levels using JC1 and MitoSox probe staining, respectively. The flow cytometry results showed that SP@NNPm significantly improved the ΔψM and reduced mitochondrial ROS production (Figure 6C,D). The TBHP‐induced increases in the mRNA expression and secretion of SASP molecules, including IL6, IL1β, and tumor necrosis factor‐alpha (TNFα), were significantly reduced in the SP@NNPm group (Figure 6E). Western blotting revealed that SP@NNPm significantly decreased the TBHP‐induced p21 and p16 while increasing the expression of the PGC1α targets NRF2 and TFAM (Figure 6F; Figure S4A, Supporting Information). To assess whether the nanoparticles' senomorphic effects could mitigate the cellular dysfunction of senescent NP cells, we examined the expression of extracellular matrix metabolism proteins. RT–qPCR and immunofluorescence staining demonstrated that compared with the other nanoparticles, SP@NNPm significantly restored the TBHP‐induced decrease in the synthesis of Aggrecan and COL2A1 while reducing the TBHP‐induced increase in the levels of the matrix‐degrading enzymes matrix metallopeptidase (MMP13) and a disintegrin and metalloproteinase with thrombospondin motifs 5 (ADAMTS5) (Figure 6G–I; Figure S4B, Supporting Information). Taken together, these results demonstrate that SP@NNPm effectively alleviates oxidative stress‐induced cellular senescence and NP cell dysfunction.

### 2.6 SP@NNPm Protects NP Cells from SASP‐Mediated Paracrine Effects

**Figure 7:**
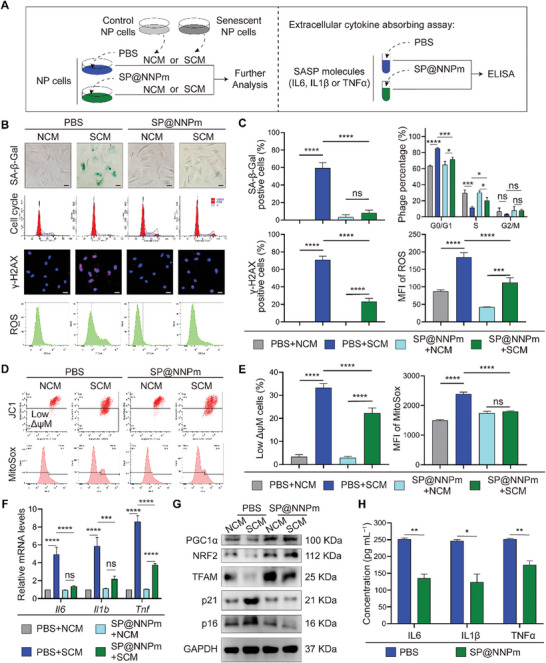
SP@NNPm inhibits SASP‐mediated paracrine effects. A) Experimental design diagram for investigation of the impact of SP@NNPm on SASP‐mediated paracrine effects (left) and extracellular cytokine absorbing assay (right). Conditioned medium was harvested from control or senescent NP cells and cocultured with NP cells in the presence of 10 µg^ ^mL^−1^ SP@NNPm or not. B,C) Representative images (B) and statistical analysis (C, *n* = 3) for SA‐β‐Gal staining, cell cycle assays, γ‐H2AX immunofluorescence staining, and ROS staining of NP cells treated with or without SCM or SP@NNPm. Black bar = 50 µm; White bar = 25 µm. Data are presented as the mean ± SD, ns, no significance, ^*^ *p* < 0.05, ^***^ *p* < 0.001, and ^****^ *p* < 0.0001 between groups. D,E) Representative flow cytometry plots (D) and statistical analysis (E, *n* = 3) for JC1 staining and MitoSox staining of NP cells treated with or without SCM or SP@NNPm. Data are presented as the mean ± SD, ns, no significance, ^****^ *p* < 0.0001 between groups. F) Statistical analysis (*n* = 3) for *Il6*, *Il1b*, and *Tnf* mRNA levels in NP cells treated with or without SCM or SP@NNPm. Data are presented as the mean ± SD, ns, no significance, ^***^ *p* < 0.001, ^****^ *p* < 0.0001 between groups. G) Representative Western blotting plots showing the levels of PGC1α, p21, p16, NRF2, and TFAM in NP cells treated with or without SCM or SP@NNPm. H) Statistical analysis (*n* = 3) for IL6, IL1β, and TNFα in extracellular cytokine absorbing assays treated with or without SP@NNPm. Data are presented as the mean ± SD, ns, no significance, ^*^ *p* < 0.05, ^**^ *p* < 0.01 between groups. NCM, conditioned medium of control (normal) NP cells; SCM, conditioned medium of senescent NP cells; SASP, senescence‐associated secretory phenotype; PBS, phosphate buffered saline; ELISA, enzyme‐linked immunosorbent assay; MFI, mean fluorescence intensity; SP, the PGC1α inducer‐loaded SiO_2_; SP@NPm, SP coated with NP cell membranes; SP@NNPm, SP coated with NKG2D‐overexpressing NP cell membranes.

The SASP in senescent cells can induce nearby normal cells to undergo senescence, thereby amplifying senescence.^[^ ^12^ ^]^ To investigate the effect of the nanoparticles on SASP‐mediated paracrine effects, we harvested conditioned medium from normal or senescent (NCM or SCM) cells and cocultured them with NP cells in the presence of SP@NNPm (**Figure** **7**A). Senescence‐related assays revealed that SP@NNPm significantly reduced SCM‐induced SA‐β‐Gal positivity, cell cycle arrest, γ‐H2AX positivity, and ROS accumulation (Figure 7B,C). Flow cytometry of JC1 and MitoSox staining demonstrated that SP@NNPm significantly improved the ΔψM and decreased mitochondrial ROS production in NP cells (Figure 7D,E). Moreover, the SCM‐induced increases in the mRNA levels of IL6, IL1β, and TNFα were significantly decreased by the SP@NNPm treatment (Figure 7F). Western blotting suggested that SP@NNPm significantly reduced the SCM‐induced increases in p21 and p16 while promoting the expression of NRF2 and TFAM (Figure 7G; Figure S4C, Supporting Information). Furthermore, we mixed SP@NNPm with SASP‐associated molecules and evaluated the remaining molecules by enzyme‐linked immunosorbent assay after removing the nanoparticles by centrifugation to assess the binding affinity of SP@NNPm to SASP‐associated molecules (Figure 7A). Only ≈50% of IL6, IL1β, and TNFα remained after coculture with SP@NNPm, indicating the excellent capacity of SP@NNPm to adsorb SASP‐associated molecules (Figure 7H). These results demonstrate that SP@NNPm effectively mitigates the paracrine effects of the SASP.

### 2.7 In Vivo Antagonism of IVDD by SP@NNPm

**Figure 8:**
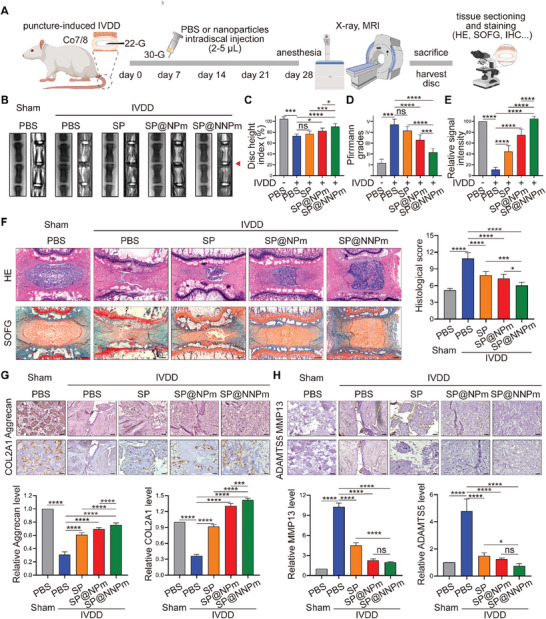
SP@NNPm effectively alleviates puncture‐induced IVDD. A) Experimental design diagram for investigation of the impact on puncture‐induced IVDD by nanoparticles. On the seventh day after surgery, IVDD rats were subjected to intradiscal injection (2–5 µL) of nanoparticles or PBS. As a control, rats in the sham group (only skin incision without puncture) were injected with PBS (5 µL). The dose of SP cores in nanoparticles was standardized as 0.6 mg^ ^kg^−1^ with the concentration of SP as 50 mg^ ^mL^−1^. Icons were created with BioRender.com. B) Representative X‐ray images and T2‐MRI images of discs from sham or IVDD rats. Red triangles indicate the coccyx disc level (Co7/8). C) Statistical analysis (*n* = 7 rats per group) of the disc height index. Data are presented as the mean ± SD, ns, no significance, ^*^ *p* < 0.05, ^***^ *p* < 0.001, and ^****^ *p* < 0.0001 between groups. D,E) Statistical analysis (*n* = 7 rats per group) of the Pfirmann grades (D) and relative signal intensity (E) of NP tissues. Data are presented as the mean ± SD, ns, no significance, ^***^ *p* < 0.001, ^****^ *p* < 0.0001 between groups. F) Representative images of HE and SOFG staining and statistical analysis (*n* = 7 rats per group) of the histological scores. Bar = 800 µm. Data are presented as the mean ± SD, ^*^ *p* < 0.05, ^***^ *p* < 0.001, and ^****^ *p* < 0.0001 between groups. G,H) Representative immunohistochemical staining images (upper) and statistical analysis (lower, *n* = 5 discs per group) for Aggrecan, COL2A1 (G), MMP13, and ADAMTS5 (H) in NP tissues. Bar = 200 µm. Data are presented as the mean ± SD, ns, no significance, ^*^ *p* < 0.05, ^***^ *p* < 0.001, ^****^ *p* < 0.0001 between groups. PBS, phosphate buffered saline; MRI, Magnetic resonance imaging; HE, hematoxylin and eosin; SOFG, safranin O fast green; IHC, immunohistochemistry; SP, the PGC1α inducer‐loaded SiO_2_; SP@NPm, SP coated with NP cell membranes; SP@NNPm, SP coated with NKG2D‐overexpressing NP cell membranes.

To evaluate the therapeutic efficacy of the nanoparticles, we established a puncture‐induced IVDD model on rat coccyx disc Co7/8 and administered intradiscal injections of the nanoparticles on the seventh day after surgery (**Figure** **8**A). X‐ray imaging revealed that SP@NNPm exhibited the most obvious reduction in the puncture‐induced decrease in disc height (Figure 8B,C). Moreover, magnetic resonance imaging analysis revealed a significant decrease in the Pfirmann grade and a significant increase in the water content in the SP@NNPm group compared to those in the other IVDD groups (Figure 8B,D,E). HE and safranin O fast green staining revealed that compared to treatment with other nanoparticles, treatment with SP@NNPm significantly alleviated the puncture‐induced morphological changes, as evidenced by the restoration of the volume and vacuolated‐like matrix in the NP and decreased histological scores (Figure 8F). Additionally, immunohistochemical staining demonstrated that SP@NNPm significantly restored the levels of the matrix proteins Aggrecan and COL2A1 while decreasing the levels of MMP13 and ADAMTS5, indicating the alleviation of matrix metabolism disruption in degenerative NP tissues (Figure 8G,H). These results demonstrate that SP@NNPm effectively alleviates puncture‐induced IVDD.

### 2.8 SP@NNPm Inhibits Cellular Senescence and Redox Imbalance by Activating the PGC1α‒NRF2/TFAM Pathway In Vivo

**Figure 9:**
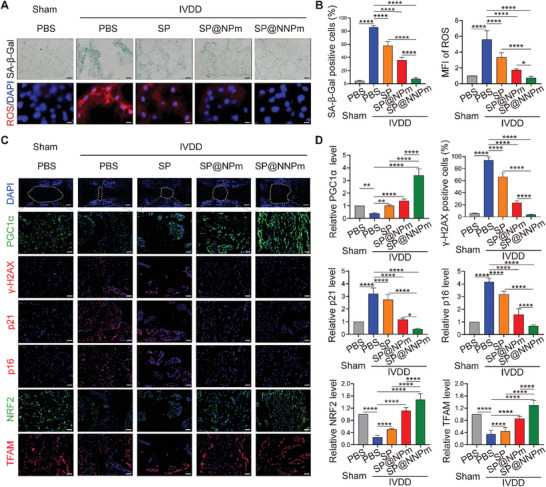
SP@NNPm alleviates cellular senescence and redox imbalance by inducing the PGC1α‒NRF2/TFAM pathway in vivo. A) Representative plots for SA‐β‐Gal staining (upper) and ROS staining (lower) of NP tissues harvested from sham rats or IVDD rats that were treated with PBS, SP, SP@NPm, or SP@NNPm. Black bar = 100 µm; White bar = 25 µm. B) Statistical analysis for SA‐β‐Gal staining and ROS staining of NP tissues (*n* = 5 discs per group). Data are presented as the mean ± SD, ^*^ *p* < 0.05, ^****^ *p* < 0.0001 between groups. C,D) Representative immunofluorescence staining plot (C) and statistical analysis (D) for the PGC1α, γ‐H2AX, p21, p16, NRF2, and TFAM level in NP tissues (*n* = 5 discs per group). Bar in the first row = 800 µm; Bar in other rows = 200 µm. Data are presented as the mean ± SD, ^*^ *p *< 0.05, ^**^ *p *< 0.01, and ^****^ *p *< 0.0001 between groups. PBS, phosphate buffered saline; MFI, mean fluorescence intensity; SP, the PGC1α inducer‐loaded SiO_2_; SP@NPm, SP coated with NP cell membranes; SP@NNPm, SP coated with NKG2D‐overexpressing NP cell membranes.

To investigate the in vivo effects of the nanoparticles on senescence, we collected fresh NP tissues from sham rats or IVDD rats that were treated with PBS, SP, SP@NPm, or SP@NNPm. SA‐β‐Gal staining indicated that the number of puncture‐induced SA‐β‐Gal positive NP cells was significantly lower in the SP@NNPm group than in the other IVDD groups (**Figure** **9**A,B). Moreover, staining with the ROS probe dihydroethidium revealed that SP@NNPm significantly mitigated the increase in the ROS levels of degenerative NP tissues from rats with puncture‐induced IVDD (Figure 9A,B). Immunofluorescence staining of senescence‐related markers suggested that SP@NNPm significantly reduced the levels of p21 and p16 and the percentage of γ‐H2AX‐positive cells in degenerative NP tissue (Figure 9C,D). Furthermore, SP@NNPm treatment significantly increased the levels of PGC1α, NRF2, and TFAM levels, as evidenced by the immunofluorescence staining assays (Figure 9C,D). Taken together, these results suggest that SP@NNPm significantly activates the PGC1α–NRF2/TFAM pathway, thereby inhibiting redox imbalance and cellular senescence during IVDD.

## 3 Discussion

Here, a biomimetic targeted nanoplatform, SP@NNPm, was developed to deliver a Pi with dual‐targeting specificity for senescent NP cells. On the one hand, by mimicking the surface properties of homologous cells, the cell membranes provide nanoparticles with multiple beneficial characteristics, such as excellent biocompatibility and enhanced selectivity for specific cell types.^[^ ^23^ ^]^ On the other hand, NKG2D is a crucial immunoreceptor that mediates immune cell recognition of senescent cells by binding to NKG2DLs, and its incorporation into immune cells can target senescent cells more precisely.^[^ ^31^, ^33^ ^]^ The expression of NKG2DLs (MICB and RAET1E) increased in senescent NP cells during disc degeneration; inspired by these findings, we first introduced NKG2D into a homologous cell membrane coating to enhance the uptake of nanoparticles by senescent NP cells. Following biocompatibility assessments, the PGC1α‐inducing ability of the nanoparticles was confirmed by increased expression levels of PGC1α and its target genes NRF2 and TFAM, resulting in enhanced mitochondrial biogenesis. SP@NNPm effectively alleviated oxidative stress‐induced senescence and inhibited SASP‐mediated paracrine effects, thus alleviating puncture‐induced IVDD.

Nanoparticle‐based delivery platforms are promising solutions to the bottlenecks of conventional therapies and are designed to enhance the effectiveness of drugs by precisely controlling their release profiles and pharmacokinetics.^[^ ^41^ ^]^ However, the ability of nanoparticles to deliver therapeutic agents to senescent cells remains a substantial limitation, with challenges including particle aggregation, potential toxicity, and weak targeting capabilities.^[^ ^42^ ^]^ Here, we present a membrane‐coated drug‐delivering strategy as a promising solution for targeting cellular senescence. SiO_2_ nanoparticles are easily synthesized nanocarriers with mesopores measuring 2–3 nm that are capable of adsorbing substances on the surface charge of their mesopores.^[^ ^43^, ^44^ ^]^ We found that these nanoparticles could accumulate in acidic lysosomes and undergo pH‐responsive drug release, which makes them suitable to deliver drugs for senescent cells with lower intracellular pH or acidic environments in degenerative intervertebral discs.^[^ ^34^, ^45^, ^46^ ^]^ Previous studies demonstrated that the adsorption and desorption dynamics of nanoparticles are dependent on the nanoparticles’ properties including particle size and functionalization.^[^ ^47^ ^]^ Membrane coating can functionalize nanoparticles for effective targeting and well biocompatibility by changing the internalization mechanism.^[^ ^48^ ^]^ Our data suggested that the membrane‐coated SiO_2_ nanoparticles displayed average sizes less than 200 nm, retained the membrane protein profile and typical cell membrane markers such as CD24 and CD155, and could be selectively internalized by NP cells. Consistently, recent studies reported that membrane coating confers nanoparticles with homologous recognition and enhanced internalization by chondrocytes, which is dependent on vesicle targeting proteins‐mediated membrane fusion and clathrin‐mediated endocytosis and micropinocytosis.^[^ ^27^, ^49^ ^]^ Furthermore, the membrane‐coated nanoparticles could adsorb SASP‐associated molecules, including IL6, IL1β, and TNFα, indicating that the membrane coating may maintain the binding abilities of these cytokines by retaining the receptors expressed on NP cell membranes, such as IL6R, IL1R, and TNFR.^[^ ^50^ ^]^ Therefore, this membrane coating strategy appears to be a suitable approach for targeting NP cells and modulating the senescence process in degenerative discs.

The NKG2D receptor and its ligands play a crucial role in immune surveillance and the clearance of senescent cells.^[^ ^31^, ^33^ ^]^ In this study, we observed that the ligands MICB and RAET1E increased in the senescent NP cells, consistent with the impaired intercellular communication and the upregulated NKG2D–NKG2DL interaction levels during puncture‐induced IVDD. Indeed, the expression of NKG2DLs and their presence on the cell surface are regulated by senescence‐associated transcription factors, including p53 and NF‐κB.^[^ ^32^, ^51^ ^]^ Previous studies showed that NKG2DLs could be consistently upregulated following induction of replicative senescence and several stresses‐induced senescence.^[^ ^31^ ^]^ Given the existence and important roles of multiple types of senescence in IVDD, this study presented a promising senescence‐targeting strategy based on the NKG2D–NKG2DL interaction.^[^ ^52^, ^53^ ^]^ Overexpression of NKG2D on the NP cell membrane significantly enhanced the uptake of membrane‐coated nanoparticles by senescent NP cells, suggesting the successful application of the senescence‐targeting strategy. Recently, NKG2D‐CAR‐T cells with CD3ζ‐modified NKG2D exhibited well‐targeting efficiency for cellular senescence or senescence‐associated immunotherapy.^[^ ^33^, ^54^, ^55^ ^]^ However, the cytotoxic activities of natural killer or T cells could be impaired by the potential release of NKG2DLs or immunosurveillance inhibitors from senescent cells, which may limit the application of NKG2D‐based cell therapy.^[^ ^56^ ^]^ Our data suggested that nanoparticles coated with NKG2D‐overexpressing membranes exerted significant senomorphic effects on oxidative stress, ROS or SASP‐induced cell senescence, and disc degeneration in vivo. Thus, this membrane coating strategy involving the NKG2D–NKG2DL interaction provides an alternative solution for targeting senescent cells and enhances the clinical applicability of senescence‐associated therapeutics.

PGC1α is a transcriptional coactivator involved in mitochondrial biogenesis and the antioxidant response.^[^ ^17^ ^]^ Our study revealed that SP@NNPm could effectively deliver ZLN005 to induce PGC1α expression, thereby significantly enhancing mitochondrial biogenesis, maintaining ΔψM, and alleviating mitochondrial ROS production in NP cells undergoing senescence. Mechanistically, our results showed that PGC1α upregulation increased the levels of NRF2 and TFAM in senescent NP cells and degenerative NP tissues. Indeed, previous research suggested that PGC1α could directly interact with several mitochondrial‐regulatory transcription factors, including NRF1/NRF2.^[^ ^57^ ^]^ The PGC1α–NRF2 pathway can promote the expression of the mitochondrial respiratory subunits.^[^ ^58^ ^]^ Moreover, numerous studies have shown that NRF2 plays a protective role in disc cells by regulating the expression of antioxidant stress genes.^[^ ^59^ ^]^ In addition, TFAM, the major driver of mtDNA packaging and transcription, can be regulated by the PGC1α–NRF1 pathway to control mitochondrial biogenesis.^[^ ^57^, ^60^ ^]^ Taken together, our in vitro and in vivo findings suggested that SP@NNPm could be an effective intervention for mitochondrial quality control in cell senescence and redox imbalance in IVDD.

## 4 Conclusion

A biomimetic dual‐targeting platform was synthesized based on homologous cell membrane fusion and the NKG2D–NKG2DL interaction. The resulting nanoparticles could effectively target senescent NP cells and activate the PGC1α–NRF2/TFAM pathway to counteract oxidative stress and SASP‐induced senescence, thereby alleviating disc degeneration. Biomimetically targeted drug delivery strategies hold promise for treating IVDD.

## 5 Experimental Section

### Cell Isolation and Treatment

All the animals in this study were obtained from the Experimental Animal Center of Tongji Medical College. The animal experiments were conducted in strict compliance with the ethical standards and protocols established by the Animal Care and Use Committee of Tongji Medical College (ethics approval number: [2023] IACUC Number: 3421). Primary NP cells and annulus fibrosus cells were isolated following established protocols.^[^ ^61^ ^]^ Cartilage endplate cells were obtained through consecutive enzymatic digestion. In brief, male Sprague‐Dawley rats (250–300 g) were anesthetized, sterilized, and dissected to expose the spine. The harvested tissue was cut into blocks (less than 1 mm^3^) and then subjected to digestion with collagenase type II (0.25%, Sigma–Aldrich, Missouri, USA) at 37 °C for 20 min. After centrifugation, collagenase type I (0.2%, Sigma–Aldrich) was added for further digestion at 37 °C for 1 h. After washing, the cells were resuspended in Dulbecco's modified Eagle's medium/F12 supplemented with fetal bovine serum (10%, Thermo Fisher Scientific, Massachusetts, USA) and penicillin/streptomycin (1%, Thermo Fisher Scientific) and cultured in an incubator at 37 °C and 5% CO_2_. Unless specified, second‐generation cells were used for further experiments.

Cellular senescence was induced as previously described.^[^ ^62^, ^63^ ^]^ In brief, NP cells were treated with TBHP (70 µmol^ ^L^−1^, Sigma–Aldrich) for 3 h to induce sublethal oxidative stress, followed by further culture in fresh medium containing serum for 21 h. NP cells treated with an equal amount of PBS (Thermo Fisher Scientific) in the same condition were used as control NP cells. The optimal exposure time and concentration were determined by propidium iodide staining and cumulative population doubling level assays (more details are provided in the supplemental information). TBHP treatment was repeated at the same time on the next day to harvest a high proportion of senescent NP cells.^[^ ^36^ ^]^ After treatment, NP cells were digested using trypsin (0.25%)‐EDTA solution (Thermo Fisher Scientific), washed twice using PBS, and harvested or seeded for the following detection or experiments. For conditioned medium collection, control NP cells and senescent NP cells were seeded separately and cultured in a serum‐free culture medium for 24 h. For the SASP‐paracrine model, the conditioned medium was subjected to filtration using a 0.2 µm filter and centrifugation at 2000 rpm for 10 min.^[^ ^64^ ^]^ Then, supernatants were harvested and used in the coculture experiments.

For NKG2D overexpression, rat NKG2D full‐length (≈650 bp) plasmids (Sino Biological Inc., China) were transfected into NP cells using Lipofectamine 3000 reagent (Thermo Fisher Scientific) according to the manufacturer's instructions. After transfection, NKG2D expression in NP cells was confirmed using the RT‒qPCR, Western blotting, and immunofluorescence staining assays (more details are provided in Supporting Information). Additionally, NP cells transfected with vector plasmids were used as control.

### Cell Membrane Preparation

Cell membranes were generated as previously described.^[^ ^65^ ^]^ NP cells were harvested, washed with PBS, and resuspended in PBS with a protease inhibitor cocktail and a phosphatase inhibitor mixture (Sigma–Aldrich). To induce cell rupture, the mixture was sonicated in an ice bath using an SFX250 Sonifier (Branson Ultrasonics, Connecticut, USA). Then, the cell homogenate was centrifuged at 2000 rpm, 4 °C for 5 min. The supernatant was continuously centrifuged at 14 000 rpm, 4 °C for 30 min and ultracentrifugated at 100 000 *g*, 4 °C for 60 min. Finally, the precipitate was collected, resuspended, and stored at −80 °C for future use.

### Preparation and Characterization of Nanoparticles

Spherical nanoparticles with diameters of ≈80–100 nm were obtained through a silicon dioxide condensation reaction catalyzed by L‐arginine.^[^ ^66^ ^]^ Mesoporous silica nanoparticles were synthesized using a hydrothermal method as previously described.^[^ ^67^ ^]^ After size selection, the resulting SiO_2_ nanoparticles were washed with deionized water and stored at 4 °C. For nanoparticle labeling, FITC‐labeled silica nanoparticles were synthesized by doping FITC‐linked (3‐aminopropyl)‐triethoxysilane conjugates as previously described.^[^ ^66^, ^68^ ^]^

For drug loading, ZLN005 (10 mg MedChemExpress, New Jersey, USA) was dissolved in dimethyl sulfoxide (1 mL) and added to a PBS solution containing mesoporous SiO_2_. Then, the mixture was ultrasonicated using an SFX250 Sonifier (Branson Ultrasonics) in an ice bath (sonication for 5 min and rest for 2 min, 3 cycles), and subjected to stirring for 8 h to precipitate Pi on the mesoporous surface. Then, the resulting nanoparticles were purified and concentrated using an ultrafiltration filter with Ultracel‐10 kDa membrane (Millipore, Massachusetts, USA) to remove DMSO. Cell membrane coating was performed using a HandExtruder‐1 mL extruder (Genizer, California, USA). In brief, the cell membranes from NP cells were extruded through porous polycarbonate membranes with pore sizes of 1000 and 800 nm to obtain vesicles of uniform size. Then, these harvested vesicles were mixed with nanoparticles at a mass ratio of 1:1 and passed through a polycarbonate membrane with a pore size of 600 nm. The coextrusion process was repeated at least 50 times. The coated nanoparticles were subsequently concentrated by freeze‐drying using a FreeZone freeze dryer (Labconco, Missouri, USA) and stored at –80 °C for future use.

For the structural analysis, the nanoparticles were examined by scanning electron microscopy at 200 kV using an FEI Talos F200X instrument (Thermo Fisher Scientific). The morphology of the membrane‐coated nanoparticles was examined by transmission electron microscopy using an HT7800 device (HITACHI, Japan) after negative staining with phosphotungstic acid (1%). The hydrodynamic size and zeta potential were determined using a Zetasizer Nano ZS90 dynamic light scattering detector (Malvern Panalytical, UK). UV spectroscopy using a NanoDrop 2000/2000c instrument (Thermo Fisher Scientific) was used to determine the peak area of the Pi at 300–310 nm as previously described.^[^ ^69^ ^]^ Encapsulation efficiency for membraned‐coated SP was determined by the red/green fluorescence positivity ratio of DiD‐labeling membrane with FITC‐doped SiO_2_ using an LSM 780 confocal microscope (Zeiss, Germany).

### Drug Loading and Release Assessment of Nanoparticles

Drug‐loading assays were conducted using inputs of ZLN005 (5 mg^ ^mL^−1^) as the Pi dissolved in dimethyl sulfoxide. The prepared nanoparticle dispersions were centrifugated at 14 000 rpm for 5 min and then resuspended in PBS to determine the drug's concentration. After detecting drug peak areas by UV spectroscopy, the drug's concentration and mass were determined based on a standard curve and known sample quantities. The mass of nanoparticles was determined using an electronic analytical balance (Sartorius, Germany) after lyophilization. Then, the drug loading statistics were calculated according to the formulas:

(1) $$loading\;content\;\;\left(wt\%\right)=\frac{W_{1}}{W_{2}\;}\;\times 100\%$$

(2) $$loading\;efficiency\;\;\left(wt\%\right)=\frac{W_{1}}{W_{3}}\;\times 100\%$$

where *W*_1_ represents the mass of the loaded drug, *W*_2_ represents the mass of drug‐loaded nanoparticles, and *W*_3_ represents the mass of the feeding drug.

For the drug release experiments of the nanoparticles, SP dispersions (1 mL, containing 2 mg of Pi) were added with PBS (1 mL) of different pH values (pH = 7.5–4.5), suspended in 5‐mL centrifuge tubes, and subjected to agitation at 37 °C. Equal volumes of the supernatant (500 µL) were collected from the centrifuge tubes at different time points (1, 2, 4, 8, 12, 24, and 48 h) and replaced with an equal volume of fresh PBS. The drug concentrations of samples were determined by UV spectroscopy using a NanoDrop 2000/2000c instrument (Thermo Fisher Scientific). The cumulative release levels were calculated according to the formula:

(3) $$Cumulative\;release\;\;\left(\%\right)=\frac{V_{1}\sum_{i=1}^{n-1}C_{i}+V_{2}C_{n}}{W_{0}}\;\times 100\%$$

where *W*_0_ represents the primary mass of Pi in SP dispersions (2 mg), *V*_1_ represents the sampling volume at each point (500 µL), *V*_2_ represents the overall volume of the release medium (1 mL), *C*_i_ represents the concentration of Pi measured at each sampling time points *i* (*i* = 1–n).

### Targeting Assessment of Nanoparticles on Disc Cells

The targeting ability of the nanoparticles was assessed through uptake assays using flow cytometry and confocal microscopy. For cell‐specific targeting assays, disc cells were labeled with different dyes, including DiI, DiD, or Hoechst 33 342 (Thermo Fisher Scientific), and mixed in equal cell amounts for coculturing with SP@NNPm‐DiO (10 µg^ ^mL^−1^). For senescent cell‐targeting assays, control and senescent NP cells were seeded under the same conditions and treated with SP@NNPm‐DiO or SP@NPm‐DiO (10 µg^ ^mL^−1^). After overnight incubation, nanoparticle‐treated cells were harvested for further flow cytometry using an LSRFortessa X‐20 flow cytometer (BD, New Jersey, USA). The percentages of positive cells were compared to analyze the targeting specificity of the nanoparticles using FlowJo v10 software (BD). For cell uptake imaging, NP cells were incubated with nanoparticles based on FITC‐doped SiO_2_, LysoTracker (Thermo Fisher Scientific), and Hoechst 33 342 (Thermo Fisher Scientific). Images of live‐cell imaging were acquired using an LSM 780 confocal microscope (Zeiss).

### Statistical Analysis

Statistical and bioinformatic analyses were performed using GraphPad Prism v8 software (GraphPad Software Inc., California, USA) and R v4.2.2 software (R Core Team, Austria). Data are expressed as means ± SD. Each experiment was performed with at least three biological replicates. The normality hypothesis and the homogeneity of variance of data were determined by Shapiro's test and Levene's test before comparisons. Unless otherwise specified, statistical differences among treatment groups were assessed using unpaired two‐tailed Student's *t*‐tests (for two groups) or one‐way analysis of variance followed by the Turkey/honestly significant difference tests (for multiple groups). All statistical analyses of experimental *n* numbers and *p* values are described in the figure legends. A level of *p* < 0.05 was considered to indicate significance.

### More Methods

Detailed methods such as animal experiments, cell viability assays, and cellular or histological staining are described in Supporting Information.

## Conflict of Interest

The authors declare no conflict of interest.

## Supporting information

Supporting Information

## Data Availability

The data that support the findings of this study are openly available in Gene Expression Omnibus at https://www.ncbi.nlm.nih.gov/geo/, reference number GSE154884 and GSE211407.
