# Supplementary material for: PGC1α‐Inducing Senomorphic Nanotherapeutics Functionalized with NKG2D‐Overexpressing Cell Membranes for Intervertebral Disc Degeneration
Source: Adv Sci (Weinh). 2024 Mar 30;11(22):2400749. doi: 10.1002/advs.202400749 (PMC11165536; doi:10.1002/advs.202400749)
Supplement: Supplementary file 1 — Supporting Information [file ADVS-11-2400749-s001.pdf]

ADVANCED SCIENCE
Open Access

Supporting Information

for *Adv. Sci.*, DOI 10.1002/advs.202400749

PGC1α-Inducing Senomorphic Nanotherapeutics Functionalized with NKG2D-Overexpressing Cell Membranes for Intervertebral Disc Degeneration

Sheng Liu, Kanglu Li, Yuxin He, Sheng Chen, Wenbo Yang, Xuanzuo Chen, Shiqing Feng, Liming Xiong, Yizhong Peng* and Zengwu Shao**

Supporting Information

PGC1α-Inducing Senomorphic Nanotherapeutics Functionalized with NKG2D-Overexpressing Cell Membranes for Intervertebral Disc Degeneration

Sheng Liu, Kanglu Li, Yuxin He, Sheng Chen, Wenbo Yang, Xuanzuo Chen, Shiqing Feng, Liming Xiong, Yizhong Peng*, Zengwu Shao**

1. Supplementary Methods

1.1. Animal model and assessment of disc degeneration

Male SD rats (300–350 g) were used to establish an intervertebral disc degeneration (IVDD) model via disc puncture.[1] After anesthesia, local disinfection, and tail skin microincision, a 22-gauge needle was inserted at a depth of 5 mm into the caudal (C) disc C7/8, which was located by manual palpation and confirmed by radiograph, and held for 30 s to induce the rat IVDD model. On the seventh day after surgery, rats were randomly divided into four groups: phosphate-buffered saline (PBS), PGC1α inducer (Pi)-loaded SiO_2 nanoparticles (SP), SP coated with nucleus pulposus (NP) cell membrane (SP@NPm), and SP coated with NKG2D-overexpressing NP cell membrane (SP@NNPm). Rats in the Sham group (only skin incision without puncture) were injected with PBS and used as the control group. Each group included 7 rats. The concentrations of SP in the SP, SP@NPm, and SP@NNPm were 50 mg mL^{-1}, and the dose of SP in these groups was standardized to 0.6 mg kg^{-1}. For each group, PBS, SP, SP@NPm, or SP@NNPm dispersions (2-5 μL) were injected into the NP using a micro syringe attached to a 30-gauge needle (Hamilton, Switzerland).

On the 28th day after surgery, X-ray and magnetic resonance imaging (MRI) were conducted under anesthesia to assess disc degeneration using an *in vivo* imaging system (BRUKER, Massachusetts, USA) and a 3.0 T MRI scanner (GE, Massachusetts, USA). For acquiring the X-ray images, the parameters were set as follows: X-ray filter = 0.4 mm; camera aperture (f-stop) = 0.95, shutter (focal plane) = -7.00 mm, and expose time = 30 s. Then, the images were analyzed using ImageJ v1.52 software (NIH, Maryland, USA) to measure the intervertebral disc height obtained from the anterior, middle, and posterior regions. The disc height index was defined as the ratio of the average disc height to the average height of adjacent vertebrae and

was adjusted by the preoperative value.[2] For acquiring the T2-weighted MRI images, pulse sequence parameters were set as follows: repetition time = 2000 ms, echo time = 71.0 ms, slice thickness = 2.0 mm, slice orientation = sagittal, sequence type = turbo spin-echo. The disc degeneration grading was performed by two blinded researchers according to the Pfirrmann grading system.[3] The signal intensity of the NP was measured using ImageJ v1.52 to calculate the relative water content (the T2-weighted signal intensity ratio of the IVDD group to that of the sham group).

The discs were collected following euthanasia for histological staining. The harvested disc tissues were fixed using a paraformaldehyde (4%) solution (Servicebio, China) and decalcified using an ethylenediaminetetraacetic acid (10%) solution (Servicebio). Then, the tissues were embedded in paraffin and subjected to 4.0-μm sectioning. The slides were stained with hematoxylin and eosin (HE) or safranin O fast green (SOFG) (Solarbio, China) according to the manufacturer's instructions. The histological images were recorded using a NanoZoomer S360 digital slide scanner (Hamamatsu, Japan). Two independent researchers blindly assessed disc degeneration based on the histological score.[4]

1.2. Hemolytic assay

Red blood cells (RBCs) from male SD rats (200 g) were used to assess the hemolytic properties of the nanoparticles. After anesthesia, a syringe with heparin was used to puncture the rat heart for blood harvesting. Then, RBCs were isolated from the blood by centrifugation at 3000 rpm for 3 min. After resuspending in PBS, the RBCs were incubated with different concentrations of nanoparticles at 37°C for 12 h. Subsequently, the absorbance (optical density, OD) value of the supernatant at 541 nm was measured using a VICTOR Nivo multimode plate reader (PerkinElmer, Massachusetts, USA). RBCs suspended in distilled water were used as the positive control and RBCs suspended in PBS without nanoparticles were used as the negative control. Then, the hemolysis rate was calculated according to the formulas:

$$Hemolysis\ (\%) = \frac{OD_i - OD_0}{OD_1 - OD_0} \times 100\% \quad (1),$$

where OD_i represents the OD values of the groups treated with specific concentrations of nanoparticles, OD_1 represents the OD value of the group treated with H_2O, and OD_0 represents the OD value of the group treated with PBS.

1.3. *In vivo* toxicity assessment

Male SD rats (200 g) were used to investigate the *in vivo* toxicity of the nanoparticles. After anesthesia, the rats were given intravenous injections of 100 μL of sterile PBS or nanoparticles

(1-5 mg kg^{-1}). On the 14th day postinjection, the heart, liver, spleen, lung, and kidney were collected after euthanasia and fixed with paraformaldehyde (4%) solution (Servicebio). After embedding and sectioning, HE staining was performed for histological examination using a CX43 light microscope (Olympus, Japan).

1.4. *In vitro* cytotoxicity assessment

The *in vitro* cytotoxicity of the nanoparticles was determined using a cell counting kit-8 (CCK-8) assay (Dojindo, Japan) according to the manufacturer's instructions. In brief, NP cells were treated with nanoparticles at different concentrations (0–100 μg mL^{-1}) for 24 h. Then, the cells were incubated with the CCK-8 working solution for 2 h at 37°C in the dark. The absorbance at 450 nm (OD450nm) was measured using a VICTOR Nivo multimode plate reader (PerkinElmer).

1.5. Live/dead cell viability assay

Cell viability was determined by calcein-AM/propidium iodide staining according to the manufacturer’s instructions. After treatment, the NP cells were incubated with calcein-AM (0.2 μmol L^{-1}, Sigma–Aldrich, Missouri, USA) for 30 min and propidium iodide (Sigma–Aldrich) for 5 min in the dark. Then, the cells were observed using a CX43 fluorescence microscope (Olympus). Additionally, NP cells were harvested at specific culture time points using trypsin (0.25%)-EDTA solution (Thermo Fisher Scientific), stained with propidium iodide (Sigma–Aldrich), and measured using a Cellometer K2 cell counter (Nexcelom Bioscience, China) to determine the optimal exposure time and concentration of TBHP.

1.6. Population doubling level (PDL) assay

The growth ability of NP cells was determined by the population doubling assays as previously described.[5,6] NP cells were cultured in a fresh medium for three days. The numbers of cells at specific culture time points were determined using a Cellometer K2 cell counter (Nexcelom Bioscience) to evaluate the growth status. PDL was calculated as the $\log_2$-fold change in the cell number relative to the last measured cell number. The cumulative PDL was defined as the sum of the PDL at the indicated culture time since the initial seeding day.

1.7. Senescence-associated β-galactosidase (SA-β-Gal) staining for cell and tissue

The β-galactosidase activity in NP cells was assessed by SA-β-Gal staining. After treatment, the NP cells were fixed for 15 min at room temperature and then exposed to a working SA-β-

Gal staining solution (Beyotime, China) at pH 6.0 in a non-CO_2 incubator at 37℃ overnight. For *in vivo* staining, fresh tissue was immediately frozen in an OCT embedding medium (Solarbio) and sectioned. After rewarming, the slides were stained as described for the *in vitro* procedures and examined under a CX43 light microscope (Olympus). Five fields of view were randomly chosen for the quantitative analysis.

1.8. Cell cycle assay

Cell cycle assays were performed to analyze the proportions of the cell cycle stages. In brief, after treatment, the cells were harvested and fixed with the precooled ethanol (70%) at 4℃ overnight. Then, the cells were incubated with preprepared PI/RNase A working solution (Kirgen, China) for 30 min at room temperature in the dark. The fluorescence at an excitation wavelength of 488 nm was detected using an LSRFortessa X-20 flow cytometer (BD, New Jersey, USA) and analyzed using Modfit LT software (Verity Software House, Maine, USA).

1.9. Reactive oxygen species (ROS) detection

The *in vitro* ROS levels in NP cells were detected using dichlorofluorescein diacetate (Sigma–Aldrich) staining. After treatment, the cells were collected and resuspended in prepared dichlorofluorescein diacetate solution and incubated at 37℃ for 20 min in the dark. After washing with serum-free medium, the samples were detected at an excitation wavelength of 488 nm using an LSRFortessa X-20 flow cytometer (BD) and analyzed by FlowJo v10 software (BD). The geometric averages of the fluorescence intensities were measured to assess the ROS levels.

The *in vivo* ROS levels in NP tissues were measured using dihydroethidium (Thermo Fisher Scientific, Massachusetts, USA) staining. After treatment, freshly obtained tissues were incubated in prediluted dihydroethidium (2 μmol L^{-1}) solution for 20 min at 37℃ in the dark. Samples were washed three times with serum-free medium and quickly subjected to OCT embedding and frozen sectioning. After rewarming and coverslipping with DAPI (Sigma–Aldrich) in the dark, the slides were examined using a CX43 fluorescence microscope (Olympus). The relative fluorescence intensity of ROS was measured by ImageJ v1.52 software (NIH) for the quantitative analysis.

1.10. MitoTracker, JC1, and MitoSox staining

Mitochondrial mass, mitochondrial membrane potential, and mitochondrial superoxide levels were determined using MitoTracker, JC1, and MitoSox staining, respectively. After treatment,

cells were harvested using trypsin (0.25%)-EDTA solution (Thermo Fisher Scientific) and then incubated with MitoTracker (0.2 μmol L^{-1}, Thermo Fisher Scientific) for 30 min, JC1 (0.1 μmol L^{-1}, MedChemExpress, New Jersey, USA) for 20 min, or MitoSox (2 μmol L^{-1}, Thermo Fisher Scientific) solutions for 20 min at 37°C in the dark. Then, the stained cells were detected using a CX43 fluorescence microscope (Olympus) or analyzed using an LSRFortessa X-20 flow cytometer (BD) and FlowJo software (BD). For MitoTracker staining, fluorescence at 510 nm was recorded and the mean fluorescence intensity (MFI) was analyzed. For JC1 staining, the fluorescence at 535 nm (monomers) and 590 nm (aggregates) was recorded. The fluorescence at 590 nm was defined as mitochondrial membrane potential (ΔψM) and percentages of cells with low ΔψM were analyzed. For MitoSox staining, fluorescence at 580 nm was recorded and the mean fluorescence intensity (MFI) was analyzed.

1.11. Assays for messenger RNA (mRNA) expression and mitochondrial DNA copy number

Gene expression levels were measured using real-time quantitative polymerase chain reaction (RT–qPCR) analysis. In brief, total RNA was isolated using TRIzol® Reagent (Thermo Fisher Scientific). The mRNA expression levels were measured using the HiScript II One Step RT–qPCR SYBR Green Kit (Vazyme, China) and the CFX96 real-time PCR detection system (Bio-Rad, California, USA). Quantification was performed using the $2^{-\triangle\triangle CT}$ method, with GAPDH expression levels used as the control.[7] The primers are listed in **Supplementary Table 1**.

Mitochondrial DNA copy number was measured by quantitative polymerase chain reaction. Total DNA from NP cells was isolated using the DNeasy Blood and Tissue Kit (Qiagen, Germany), and the mtDNA-encoded mitochondrial 16S rRNA expression levels were normalized to nuclear DNA EEF1A1 using the $2^{-\triangle\triangle CT}$ method to determine the relative mtDNA content.[8] The primers are listed in **Supplementary Table 1**.

1.12. Protein extraction and Western blotting

Protein expression levels were assessed via Western blotting. In brief, proteins were extracted from cell lysates, membranes, or nanoparticles using a protein extraction kit (Beyotime), and the protein concentration was determined using a protein concentration determination (bicinchoninic acid method) kit (Beyotime). Then, protein (20 μg) was separated by sodium dodecyl-sulfate polyacrylamide gel (10% or 12.5%) electrophoresis and then transferred onto a polyvinylidene fluoride membrane (Millipore, Massachusetts, USA). The polyvinylidene fluoride membrane was blocked in skim milk (5%) at room temperature for 60 min and

incubated overnight at 4℃ with the following primary antibodies: anti-NKG2D (ab36136, 1:1000, Abcam, UK), anti-CD24 (ab179821, 1:1000, Abcam), anti-CD155 (ab205304, 1:1000, Abcam), anti-CD221 (ab182408, 1:1000, Abcam), anti-Na^{+}, K^{+} ATPase (a12405, 1:1000, ABclonal, China), anti-PGC1α (66369-1-Ig, 1:1000, Proteintech, China), anti-NRF2 (16396-1-AP, 1:1000, Proteintech), anti-TFAM (22586-1-AP, 1:1000, Proteintech), anti-p21 (sc-6246, 1:1000, Santa Cruz, Texas, USA), anti-p16 (sc-1661, 1:1000, Santa Cruz), or anti-GAPDH (60004-1-Ig, 1:1000, Proteintech) antibody. After washing with PBS, the PVDF membrane was incubated with secondary antibodies, including goat anti-mouse, goat anti-rabbit, or donkey anti-goat IgG (SA00001-1/2/3, 1:3000, Proteintech) at room temperature for 60 min. Finally, the immunoblots were visualized using an enhanced chemiluminescence kit (Biosharp, China) and a ChemiDoc imaging system (Bio-Rad), and protein quantification was performed using ImageJ v1.52 software (NIH).

1.13. Coomassie blue staining

The protein distribution pattern was determined by Coomassie blue staining. According to the manufacturer's instructions, sodium dodecyl-sulfate polyacrylamide gels were fixed after electrophoresis using a methanol and acetic acid mixture for 60 min, sensitized using glutaraldehyde, and incubated with a Coomassie blue staining solution (Biosharp) for 1–2 h. Then, the gels were washed with a prepared solution (40% ethanol, 10% acetic acid, and 50% double-distilled water) for 4 h to remove the background, and were detected using a ChemiDoc imaging system (Bio-Rad).

1.14. Enzyme-linked immunosorbent assay (ELISA) and extracellular cytokine absorbing assay

Levels of IL6, IL1β, and TNFα were quantified using the ELISA kits (E-EL-R0015/E-EL-R0012/E-EL-R2856, Elabscience, China) according to the manufacturer's directions. In brief, standard products were added to the well plate to generate a standard curve. After removing the nanoparticles by centrifugation at 14000 rpm for 5 min, culture medium or supernatants were added to the sample wells. Horseradish peroxidase-conjugated antibodies were added for 60 min at 37℃. After washing, the substrate was added for 15 min in the dark. After terminating the reaction, the absorbances at 450 nm were measured using a VICTOR Nivo plate reader (PerkinElmer), and the concentrations were calculated based on the standard curve.

For the extracellular cytokine absorbing assay, the rat recombinant proteins IL6, IL1β, or TNFα (0.5 ng mL^{-1}, PROTP20607/PROTQ63264/PROTP16599, Boster, China) were prepared.

Equal volumes of SP@NNPm (10 μg mL^{-1}) dispersions or PBS were added and subjected to incubation for 12 h. After the nanoparticles were removed by centrifugation at 14,000 rpm for 5 min, the supernatant was collected for ELISA assays. The absorbance at 450 nm was measured using a VICTOR Nivo plate reader (PerkinElmer) and the concentration of the supernatant was calculated based on the standard curve.

1.15. Immunohistochemistry

In brief, tissue sections were deparaffinized, rehydrated, and subjected to heat-induced antigen retrieval. After blocking with hydrogen peroxide (3%) solution for 10 min and donkey serum (10%, Servicebio) supplemented with Triton (0.5%, Sigma–Aldrich) for 60 min, the sections were incubated overnight at 4℃ with the following primary antibodies: anti-Aggrecan (13880-1-AP, 1:100, Proteintech), anti-COL2A1 (ab34712, 1:50, Abcam), anti-MMP13 (18165-1-AP, 1:100, Proteintech), and anti-ADAMTS5 (ab41037, 1:100, Abcam) antibody. After several washes, the sections were then incubated with secondary antibodies and a diaminobenzidine (DAB) substrate kit (Boster). Finally, the sections were counterstained and coverslipped for microscopic analysis using a CX43 fluorescence microscope (Olympus). Five random fields were chosen for the quantitative analysis.

1.16. Immunofluorescence staining

Immunofluorescence staining was used to assess the protein levels. For the *in vitro* experiment, cell-seeded slides were fixed using paraformaldehyde (4%, Servicebio). For tissue staining, slides were deparaffinized and subjected to antigen retrieval by heating. The slides were then incubated in a PBS solution containing Triton (0.5%) for permeabilization and blocked in donkey serum (10%, Servicebio) at 37℃ for 45 min. Then, the slides were incubated with primary antibodies at 4℃ overnight. The following antibodies were used: anti-NKG2D (ab36136, 1:100, Abcam), anti-PGC1α (66369-1-Ig, 1:100, Proteintech), anti-NRF2 (16396-1-AP, 1:50, Proteintech), anti-TFAM (22586-1-AP, 1:150, Proteintech), anti-γ-H2AX (ab11174, 1:100, Abcam), anti-p21 (sc-6246, 1:50, Santa Cruz), anti-p16 (sc-1661, 1:50, Santa Cruz), anti-Aggrecan (13880-1-AP, 1:100, Proteintech), anti-COL2A1 (ab34712, 1:50, Abcam), anti-MMP13 (18165-1-AP, 1:100, Proteintech), and anti-ADAMTS5 (Ab41037, 1:100, Abcam) antibody. After rewarming and washing, the slides were incubated with AF594-conjugated donkey anti-goat (A-11058, 1:500, Thermo Fisher Scientific), AF488-conjugated goat anti-rabbit (A-11008, 1:500, Thermo Fisher Scientific), AF594-conjugated goat anti-rabbit (A-11012, 1:500, Thermo Fisher Scientific), or AF594-conjugated goat anti-mouse (A-11032,

1:500, Thermo Fisher Scientific) secondary antibodies. After adding an anti-quenching sealing agent (Servicebio), the cell slides were imaged using a CX43 fluorescence microscope (Olympus). Five random fields were chosen for the quantitative analysis.

1.17. Flow cytometric analysis of NKG2D ligand expression

After treatment, control and senescent cells were harvested and directly incubated with an anti-MICB (orb1241, 1:100, Biorbyt, UK), anti-RAET1E (ab95202, 1:100, Abcam) or isotype control (ab172730/ab170191, 1:100, Abcam) antibodies for 60 min at 4℃. The cells were washed three times and incubated with PE-conjugated goat anti-rabbit IgG (A10542, 1:200, Thermo Fisher Scientific) or APC-conjugated goat anti-mouse IgG (17-4010-82, 1:200, Thermo Fisher Scientific) secondary antibody for 30 min. Fluorescence was measured with an LSRFortessa X-20 flow cytometer (BD) and analyzed using FlowJo v10 software (BD).

1.18. Bioinformatic analysis

The intercellular communication profile of rat disc cells was reanalyzed using the single-cell RNA sequencing datasets (GSE154884 and GSE211407).[9,10] The raw data were downloaded from the Gene Expression Omnibus database and mapped to the Rattus norvegicus genome v6.0 by cellranger v6.1.1 software. The Seurat v4.3.0.1 package was used to import the count matrixes and filter out the cells of poor sequencing quality (doublet cells predicted by DoubletFinder v2.0.3 or cells with fewer than 200 or more than 6,000 unique molecular identifiers or more than 20% of mitochondria-associated unique molecular identifiers). Seurat objects were merged using the Harmony v0.1.1 algorithm and subjected to unsupervised clustering with the default parameters. The Harmony embeddings were further visualized by the UMAP algorithm for dimensionality reduction. Then, the rat genes were transformed into human orthologs using the biomaRt v2.56.1 package. The four major cell types of disc cells were identified through manually curated markers and primary cell annotation information via the SingleR v 2.2.0 package.

The intercellular communication profile among these cell types was analyzed using the CellChat v1.6.1 package. According to the CellChat instructions, we used the subset ("Secreted Signaling" and "Cell–Cell Contact") of the CellChat databases and the modified parameter (type = "truncatedMean," trim = 0.05) in the computation of the communication probability/strength to explore the relatively weak but important pathways of cell–cell interactions. The interaction number and strength were predicted for all permutations of the cell types and compared among

the IVDD groups. Additionally, the interactions between NKG2D and its ligands were further evaluated using CellPhoneDB v4 software.

1.19. References

[1] Y. Peng, X. Qing, H. Lin, D. Huang, J. Li, S. Tian, S. Liu, X. Lv, K. Ma, R. Li, Z. Rao, Y. Bai, S. Chen, M. Lei, D. Quan, Z. Shao, *Bioact. Mater.* **2021**, *6*, 3541.
[2] K. Masuda, Y. Aota, C. Muehleman, Y. Imai, M. Okuma, E.J. Thonar, G.B. Andersson, H.S. An, *Spine (Phila Pa 1976)*. **2005**, *30*, 5.
[3] C.W. Pfirrmann, A. Metzdorf, M. Zanetti, J. Hodler, N. Boos, *Spine (Phila Pa 1976)*. **2001**, *26*, 1873.
[4] B. Han, K. Zhu, F.C. Li, Y.X. Xiao, J. Feng, Z.L. Shi, M. Lin, J. Wang, Q.X. Chen, *Spine (Phila Pa 1976)*. **2008**, *33*, 1925.
[5] S. Ekram, S. Khalid, F. Ramzan, A. Salim, I. Bashir, M.C. Durrieu, I. Khan, *Cartilage*. **2023**, 19476035231172154.
[6] S. Wedel, I. Martic, N. Hrapovic, S. Fabre, C.T. Madreiter-Sokolowski, T. Haller, G. Pierer, C. Ploner, P. Jansen-Durr, M. Cavinato, *Mech. Ageing Dev.* **2020**, *190*, 111318.
[7] T. Yurube, T. Takada, H. Hirata, K. Kakutani, K. Maeno, Z. Zhang, J. Yamamoto, M. Doita, M. Kurosaka, K. Nishida, *J. Orthop. Res.* **2011**, *29*, 1284.
[8] H. Fan, R. Ding, W. Liu, X. Zhang, R. Li, B. Wei, S. Su, F. Jin, C. Wei, X. He, X. Li, C. Duan, *Redox Biol.* **2021**, *40*, 101856.
[9] J. Wang, Y. Huang, L. Huang, K. Shi, J. Wang, C. Zhu, L. Li, L. Zhang, G. Feng, L. Liu, Y. Song, *Osteoarthritis Cartilage*. **2021**, *29*, 389.
[10] M. Rohanifar, S.W. Clayton, G.W.D. Easson, D.S. Patil, F. Lee, L. Jing, M.N. Barcellona, J.E. Speer, J.J. Stivers, S.Y. Tang, L.A. Setton, *Appl. Sci.-Basel*. **2022**, *12*, 8244.

2. Supplementary Figures

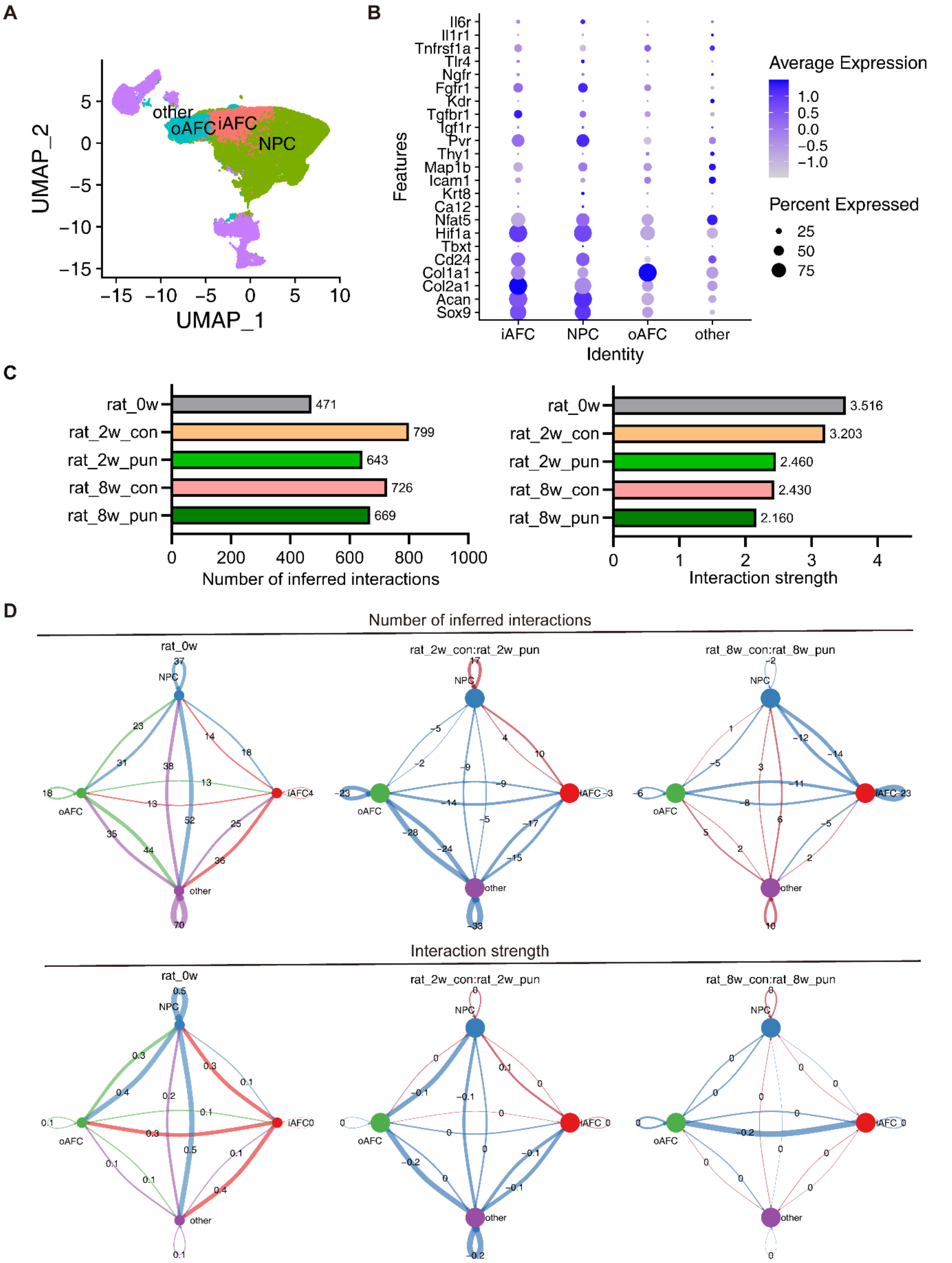

Figure S1. Impaired intercellular communication profile during rat disc degeneration. The single-cell RNA-sequencing data was merged from the datasets GSE154884 (generated from disc cells of 8-week-old rats, defined as rat_0w) and GSE211407 (generated from disc cells of sham or puncture-induced IVDD rats at 2 weeks or 8 weeks, grouped as rat_2w_con, rat_2w_pun, rat_8w_con, and rat_8w_pun). **(A)** UMAP plot for cell types. **(B)** Cell identify plot for cell type annotations. **(C)** The number (left) and strength (right) of inferred interactions in

CellChat analysis. **(D)** Plots for the number (upper) and strength (lower) of CellChat-inferred intercellular actions. The cell-cell actions were analyzed for normal status (rat_0w) and the comparisons (rat_2w_con vs rat_2w_pun; rat_8w_con vs rat_8w_pun). NPC, nucleus pulposus cell; iAFC, inner annulus fibrosus cell; oAFC, outer annulus fibrosus cell; other, other cell type.

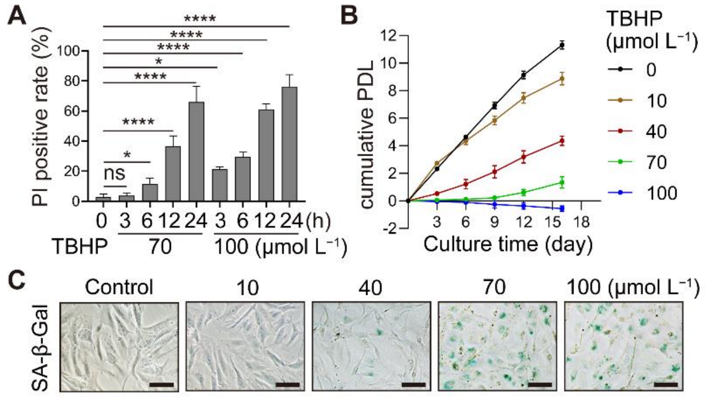

Figure S2. Optimal time and concentration for inducing NP cell senescence. (A) Barplot for PI staining assays (n = 3) on NP cells after TBHP (70 or 100 μmol L^{-1}) with different treatment times (3–24 h). The result showed that 70 μmol L^{-1} TBHP treatment for 3 h did not induce cell death. Data are presented as the mean ± SD, ns, no significance, *p < 0.05, ****p < 0.0001 between groups. **(B)** The cumulative PDL plot for NP cells after TBHP treatment at different exposure concentrations (10–100 μmol L^{-1}). The result showed that TBHP treatment with a concentration of 70 μmol L^{-1} or 100 μmol L^{-1} impaired the growth ability of NP cells. Data are presented as the mean ± SD, n = 3. **(C)** Representative SA-β-Gal staining images for NP cells exposed to TBHP treatment at different concentrations (10–100 μmol L^{-1}). Bar = 50 μm. PI, propidium iodide; PDL, population doubling level.

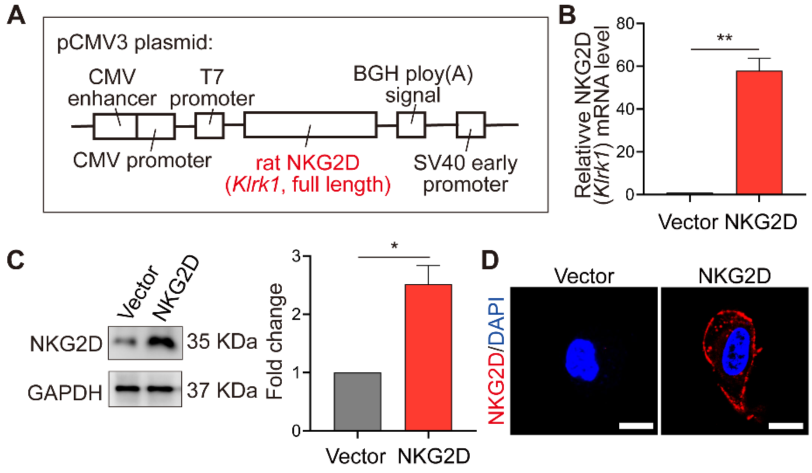

Figure S3. overexpression of NKG2D in NP cells. (A) Structural diagram of the rat NKG2D (*Klrk1*) overexpressing plasmid. The empty vector without the open reading frame sequence of NKG2D was used as the control. **(B)** Statistical analysis of the relative NKG2D mRNA *Klrk1* expression levels in NP cells transfected with the plasmids (n = 3). Data are presented as the mean ± SD, **p < 0.01 between groups (Welch's t-test). **(C)** Representative Western blotting plots and statistical analysis for NP cells transfected with the plasmids (n = 3). Data are presented as the mean ± SD, *p < 0.05, between groups. **(D)** Representative images of NKG2D immunofluorescence staining for NP cells transfected with the plasmids. Bar = 10 μm.

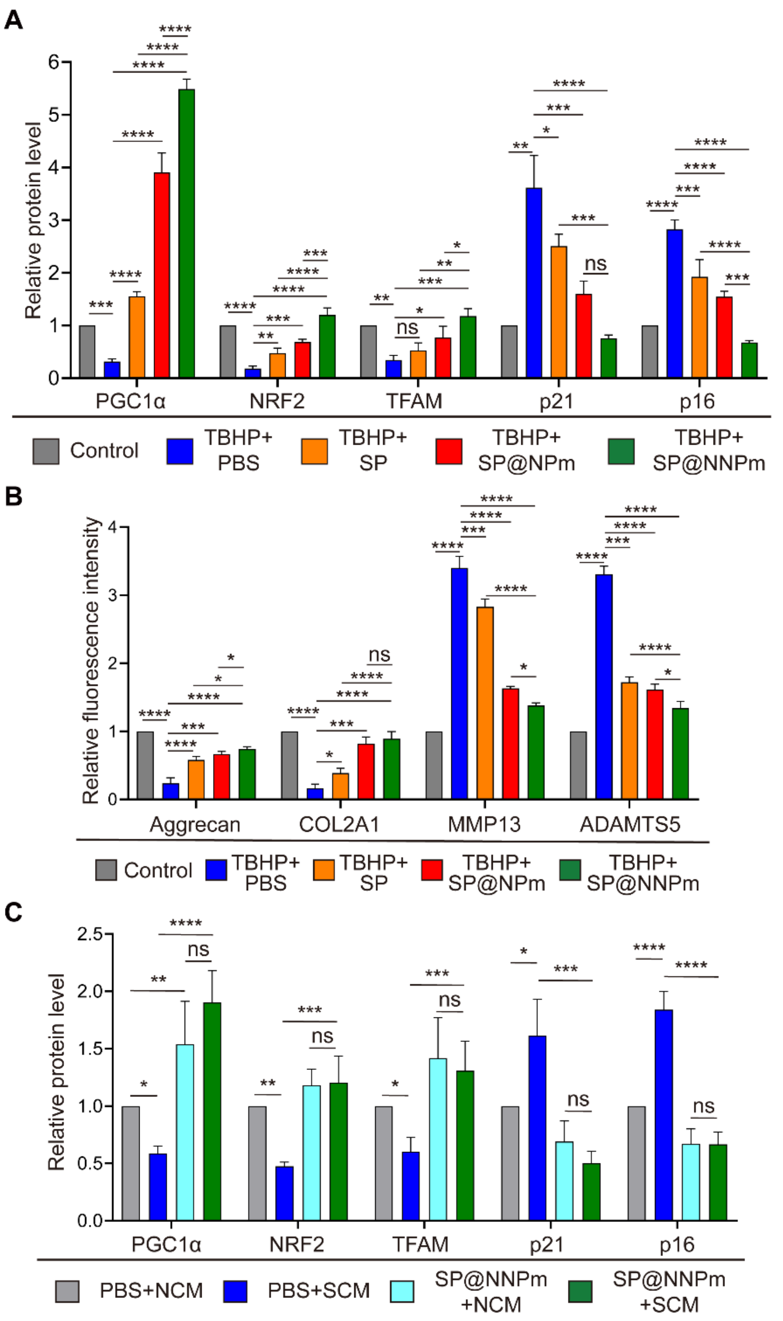

Figure S4. SP@NNPm enhances the PGC1α–NRF2/TFAM pathway and regulates cellular senescence. (A) Statistical analysis (n = 3) of Western blotting assays for the levels of PGC1α, p21, p16, NRF2, and TFAM in NP cells treated with or without 70 μmol L^{-1} TBHP or the nanoparticles. Data are presented as the mean ± SD, ns, no significance, *$p < 0.05$, **$p < 0.01$, ***$p < 0.001$, ****$p < 0.0001$ between groups. **(B)** Statistical analysis (n = 3) of immunofluorescent staining for Aggrecan, COL2A1, MMP13, and ADAMTS5 in NP cells treated with or without 70 μmol L^{-1} TBHP or the nanoparticles. Data are presented as the mean ± SD, ns, no significance, *$p <$ 0.05, ***$p < 0.001$, ****$p < 0.0001$ between groups. **(C)** Statistical analysis (n = 3) of Western blotting assays for the levels of PGC1α, p21, p16, NRF2, and TFAM in NP cells treated with or without SCM or SP@NNPm. Data are presented as the mean ± SD, ns, no significance, *$p < 0.05$, **$p < 0.01$, ***$p < 0.001$, ****$p < 0.0001$ between groups. NCM, conditioned medium of control (normal) NP cells; SCM, conditioned medium of senescent NP

cells; PBS, phosphate buffered saline; SP, the PGC1α inducer-loaded SiO_2; SP@NPm, SP coated with NP cell membranes; SP@NNPm, SP coated with NKG2D-overexpressing NP cell membranes.

3. Supplementary Table

Supplementary Table 1. Primers for RT–qPCR and mitochondrial DNA copy number assays.

Gene name (Protein name)	Type	Sequence (5' to 3')
Gapdh (GAPDH)	Forward	GGCATCGTGGAAGGGCTCAT
	Reverse	GATACATTGGGGGTAGGAACAC
Klrk1 (NKG2D)	Forward	CAACTTGAATTGCTTCCTCCAGA
	Reverse	AGAGGAGCCATCTTCCCACT
Ppargc1a (PGC1α)	Forward	AAACTTGCTAGCGGTCCTCA
	Reverse	TGTTGACAAATGCTCTTC
Nfe2l2 (NRF2)	Forward	CTATCTGCTGGTTCCCCACTGCT
	Reverse	GGCTGGCTGAATTGGGAGGA
Tfam (TFAM)	Forward	CCTCGCCTGTCAGCCTTATC
	Reverse	CGGGCTTCCTTCTCTAAGCC
Il6 (IL6)	Forward	CCTGGAGTTTGTGAAGAACAACT
	Reverse	GGAAGTTGGGGTAGGAAGGA
Tnf (TNFα)	Forward	GCCTCTTCTCATTCCTGCTCG
	Reverse	CTGATGAGAGGGAGCCCATT
Il1b (IL1β)	Forward	TGTGATGAAAGACGGCACAC
	Reverse	CTTCTTCTTTGGGTATTGTTTGG
Adamts5 (ADAMTS5)	Forward	GACAGTTCAAAGCCAAAGACC
	Reverse	TTTCCTTCGTGGCAGAGT
Mmp13 (MMP13)	Forward	TCAGGAAACCAGGTCTGGAG
	Reverse	TGACGCGAACAATACGGTTA
Col2a1 (COL2A1)	Forward	AATTCCTGGAGCCAAAGGAT
	Reverse	AGGACCAGTTGCACCTTGAG
Acan (Aggrecan)	Forward	GCAGCACAGACACTTCAGGA
	Reverse	CCCACTTTCTACAGGCAAGC
Eef1a1 (EEF1A1)	Forward	GGACTGCCACACGGCCCACATA
	Reverse	GGAGGGTAGTCAGAGAAGCTTTC
Mitochondrial 16S rRNA	Forward	CCGCAAGGGAAAGATGAAAGAC
	Reverse	TCGTTTGGTTTCGGGGTTTC
